# Neural landscape diffusion resolves conflicts between needs across time

**DOI:** 10.1038/s41586-023-06715-z

**Published:** 2023-11-08

**Authors:** Ethan B. Richman, Nicole Ticea, William E. Allen, Karl Deisseroth, Liqun Luo

**Affiliations:** 1 Neurosciences Graduate Program, Stanford University, Stanford, CA USA; 2 Department of Bioengineering, Stanford University, Stanford, CA USA; 3 Department of Biology, Stanford University, Stanford, CA USA; 4 Department of Psychiatry and Behavioral Sciences, Stanford, CA USA; 5 Howard Hughes Medical Institute, Stanford University, Stanford, CA USA; 6 Present Address: Department of Applied Physics, Stanford University, Stanford, CA USA; 7 Present Address: Society of Fellows, Harvard University, Cambridge, MA USA

**Keywords:** Neural circuits, Motivation, Statistical physics

## Abstract

Animals perform flexible goal-directed behaviours to satisfy their basic physiological needs^1–12^. However, little is known about how unitary behaviours are chosen under conflicting needs. Here we reveal principles by which the brain resolves such conflicts between needs across time. We developed an experimental paradigm in which a hungry and thirsty mouse is given free choices between equidistant food and water. We found that mice collect need-appropriate rewards by structuring their choices into persistent bouts with stochastic transitions. High-density electrophysiological recordings during this behaviour revealed distributed single neuron and neuronal population correlates of a persistent internal goal state guiding future choices of the mouse. We captured these phenomena with a mathematical model describing a global need state that noisily diffuses across a shifting energy landscape. Model simulations successfully predicted behavioural and neural data, including population neural dynamics before choice transitions and in response to optogenetic thirst stimulation. These results provide a general framework for resolving conflicts between needs across time, rooted in the emergent properties of need-dependent state persistence and noise-driven shifts between behavioural goals.

## Abstract (web summary)

Behavioural and electrophysiological studies in simultaneously thirsty and hungry mice reveal a neural basis for resolving conflicts between needs, in which choices are guided by a persistent and distributed neural goal state that undergoes spontaneous transitions between goals.

## Main

**Fig. 1:**
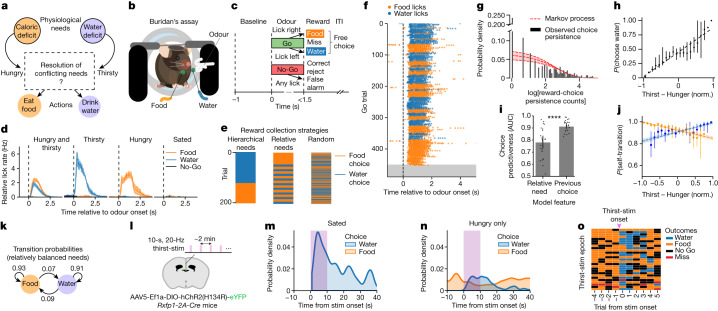
Reward choice under conflicting needs is structured by persistent behavioural states with stochastic transitions. **a**, The conceptual problem. **b**, Buridan’s assay. A food- and water-restricted mouse is head-restrained with two equally accessible reward spouts, delivering salted liquid food and water, respectively. **c**, Trial structure. Go odour indicates reward availability and No-Go odour indicates reward unavailability after a variable inter-trial interval (ITI). After Go-odour onset, mice freely choose food or water reward by licking right or left, respectively. **d**, Licking behaviour during Buridan’s assay under different restriction conditions. The *y* axis shows average lick rate at a given spout, multiplied by the fraction of licks to that spout per session. Data are mean ± s.e.m. *n* = 15 mice, 22 sessions for food and water restriction; *n* = 3 mice, 3 sessions for water or food restriction only; *n* = 2 mice, 2 sessions for no restrictions. **e**, Hypothetical reward-choice patterns under different strategies. **f**, Behavioural session showing food and water licks across trials until satiation (grey). **g**, Reward-choice persistence counts distribution for all behavioural sessions with both food and water restriction. Dashed red line indicates probability density for log[persistence counts] generated by a sticky Markov process (geometric distribution fit to data, maximum likelihood shape parameter *P* = 0.061, 95% confidence interval [0.05, 0.074]). **h**, Probability of choosing a water reward on rewarded Go trials, fit by linear regression (dashed line) to observed relative need (normalized (norm.) thirst − hunger). *R*^2^ = 0.92, slope = 0.426. Data are mean ± 95% confidence interval. The first and last two data points lack confidence intervals owing to too few data points. **i**, Prediction of current choice as a function of current needs or the most recent previous choice, based on a support vector machine model. AUC, receiver operating characteristic area under the curve. Data are mean ± 95% confidence interval. Two-sided paired *t*-test; *n* = 22 sessions, *t* = −5.89, *P* = 6.28 × 10^−6^. **j**, Self-transition probability fit by linear regression to normalized thirst − hunger. Data are mean ± 95% confidence interval. Water choice: *R*^2^ = 0.612, slope = 0.07; food choice: *R*^2^ = 0.844, slope = −0.077. **k**, Go-trial transition probability between reward choices. Probabilities are maximum likelihood estimates from trials with normalized thirst − hunger between −0.25 and 0.25. **g**–**k**, *n* = 15 mice, 22 sessions. **l**, Schematic of optogenetic activation of osmotic thirst (RXFP1^+^) neurons in the subfornical organ (green) in 10-s epochs during Buridan’s assay. **m**,**n**, Probability density (kernel density estimate) of food and water choices in Go trials as a function of optogenetic thirst stimulation (purple bars), in experiments on sated mice (**m**; *n* = 2 mice, 63 stim epochs) or on hungry-only mice (**n**; *n* = 2 mice, 69 stim epochs). **o**, Trial outcomes (colour-coded, right) surrounding each optogenetic thirst-stimulation epoch (rows; *n* = 27) from a single session on a hungry-only mouse.

Deviations from physiological homeostasis produce diverse needs, such as thirst and hunger, and drive profound changes in an animal’s behaviour^3,4^. These needs have historically been conceived as distinct forces acting on animal behaviour, with effects gated by the availability of appropriate rewards^13^. Recent studies have established neurobiological bases for detecting individual physiological imbalances and for generating goal-directed behavioural^9–12^ and neural^14^ states. Animals in nature often confront multiple co-occurring needs, yet still exhibit discrete and coherent goal-directed actions. Precisely how conflicts between needs are resolved, especially in the case of equally available rewards, has been a subject of perplexity since the time of Aristotle, who questioned whether an equally hungry and thirsty person would remain stuck between equidistant food and water^15^; later philosophers replaced the person with a donkey and popularized this quandary as ‘Buridan’s ass’^16^. Although neurobiological studies have compared the circuit and behavioural properties of thirst and hunger and their interactions, these needs have not been studied in a conflicting, moment-by-moment context^9,17–22^. We reasoned that the quandary of Buridan’s ass highlights an incomplete conceptual framework relating needs to motivated behaviour—namely, a lack of neurobiological explanation for how conflicting needs could jointly organize behaviour across time (Fig. 1a). A more complete framework for resolving conflicting needs across time should: (1) relate the intensity and salience of individual needs to behavioural choices at any given moment; (2) identify a neural basis for behavioural choices; and (3) explain the dynamics of switching between need-appropriate behaviours.

## Choice assay for conflicting needs

**Extended Data Fig. 1:**
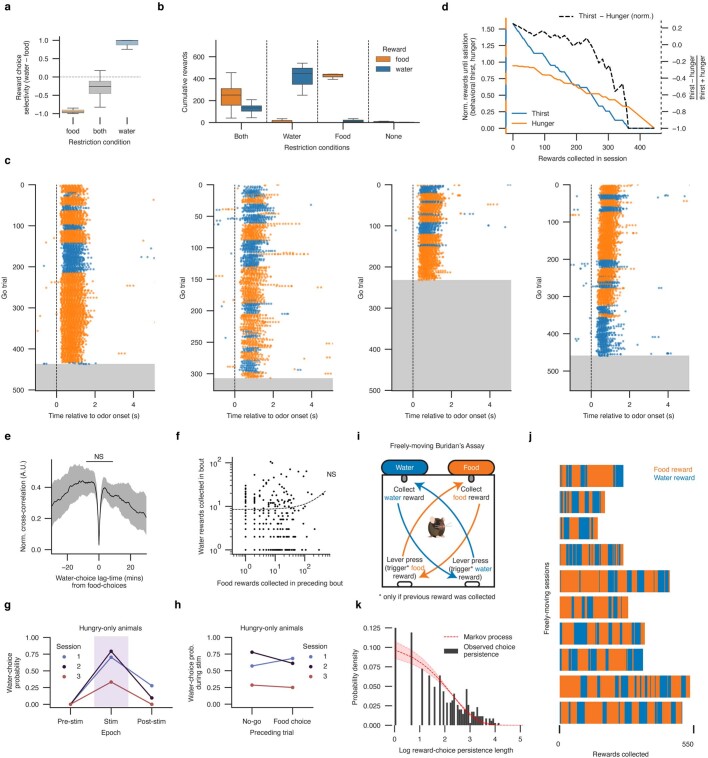
Quantification of behavior in Buridan’s assay. **a**, Choice selectivity [(water choices – food choices)/(food choices + water choices)] in Buridan’s assay under different restriction conditions. **b**, Cumulative food and water rewards collected for each restriction condition. Boxplots in **a**, **b** show data ranging from the lower quartile to the upper quartile, whiskers extend to 1.5-times the inter-quartile range. *n* = 15 mice, 22 sessions for food and water restriction, *n* = 3 mice, 3 sessions for both water restriction only and food restriction only; *n* = 2 mice, 2 sessions for no restrictions. **c**, Additional example behavior sessions. Each row shows licking behavior (food licks, orange dots; water licks, blue dots) during a trial. Grey region, sated non-responses. **d**, Example calculation of behavioral thirst, behavioral hunger, and relative need [Thirst – Hunger (norm.) = (thirst – hunger)/(thirst + hunger)] for a behavioral session. Behavioral thirst and hunger are normalized by their median values across all sessions. **e**, **f**, Food choices do not significantly induce water choices on the timescale of Buridan’s assay. **e**, Normalized cross-correlation plot indicating lag time between water choices and food choices. The normalized cross correlation is not significantly different from a zero lag-time (1-sample two-sided T-test, *t* = –1.45, *P* = 0.16). A significant positive or negative lag time would indicate that food choices induce water choices or water choices induce food choices, respectively. Mean ± 95% confidence interval, *n* = 15 mice, 22 sessions. **f**, Number of water rewards collected in a given bout (y-axis) vs. the number of food rewards collected in the previous bout (x-axis), both log-scaled. Dots, rewards in bout; dashed line, linear fit. No significant (NS) relationship was found between water rewards in a bout and the number of food rewards in a previous bout (*R*^2^ = 0.0063, *P* = 0.185). **g, h**, Additional quantification of optogenetic thirst stimulation in hungry animals during Buridan’s assay. **g**, Probability of water choices before (–20 s to –10 s before stimulation onset), during (0.5 s to 10 s after stimulation onset), or after (30 s to 40 s after stimulation onset) optogenetic stimulation, plotted for each of the three sessions. **h**, Comparison of the water-choice probability during stimulation for epochs following food choices or No-Go trials. No significant influence of previous trial outcome is found on the subsequent stimulation epoch water-choice probability (two-sided paired t-test, *t* = 0.37, *P* = 0.747). Blue and black markers indicate data from one animal; the red marker indicates data from a second animal. Each session contains 30 optogenetic stimulation epochs. **i–k**, A freely moving version of Buridan’s assay. **i**, Schematic. Hungry and thirsty mice are placed in a behavioral chamber in which incremental salted liquid food or water rewards are dispensed from spouts upon pressing of the corresponding food or water lever on the opposite wall of the chamber. Rewards must be collected from a given spout before subsequent lever presses dispense additional rewards. Spouts and their corresponding reward levers are placed diagonally across from each other in the chamber, such that the mouse must pass through the center of the chamber (equidistant from both food and water levers). There are no choice-cues in this assay: the mice are free to initiate or stop lever pressing and reward collection at any time. Mice were trained in the arena until proficient at collecting corresponding rewards following lever presses. **j**, Food (orange) and water (blue) reward collection sequences for 6 mice across 10 sessions (rows). **k**, Choice persistence length (# repeated rewards of the same kind before switching) distribution across the 10 sessions. Dashed red line and shaded region, fit geometric distribution corresponding to a sticky Markov process, mean shape parameter *P* = 0.097 ± 95% confidence interval [0.086, 0.108].

We developed an experimental paradigm that we term Buridan’s assay, in which simultaneously hungry and thirsty mice were repeatedly given a free choice between satiating one need or the other, but not both at once (Fig. 1b,c). Mice were food- and water-restricted, head-restrained and placed in front of two equally accessible reward spouts delivering water or salted liquid food (Fig. 1b). In a modified olfactory Go/No-Go paradigm^14,23^, a Go odour indicated that both food and water rewards were available; however, which reward was dispensed on a given trial depended on the mouse’s free choice, determined by the direction of their first lick (Fig. 1c). A No-Go odour indicated reward unavailability. Go-odour trials (67% frequency) were randomly interleaved with No-Go-odour trials (33% frequency). Mice learned to choose either food or water in response to the Go odour, and to withhold licking during No-Go odours and the variable inter-trial interval (Fig. 1d). After training, food- and water-restricted mice performed hundreds of trials across a behavioural session, collecting incremental food and water rewards until sated. Trained mice made need-appropriate reward choices: food-restricted mice mostly chose food rewards; water-restricted mice mostly chose water rewards; and food- and water-restricted mice chose both food and water rewards within a given session (Fig. 1d and Extended Data Fig. 1a,b).

## Persistent, stochastic choice behaviour

We next investigated what strategy an animal might pursue to resolve conflicting needs across a session. In a hierarchical needs model, mice would repeatedly choose one reward type until satiation, then switch to satiate the other need (Fig. 1e, left). In a relative needs model, mice would choose to reward the more deficient need until equality and subsequently oscillate regularly between each reward choice, subject to a fixed feedback delay to account for the time it takes for food or water ingestion to affect behaviour^7,8^ (Fig. 1e, middle). In a random model, mice would choose rewards arbitrarily until both needs were sated (Fig. 1e, right). None of these models matched our data; instead, we found that food- and water-restricted mice made highly persistent reward choices punctuated by sudden switches (Fig. 1f and Extended Data Fig. 1c), forming spontaneous reward-choice bouts. This pattern is characteristic of a Markov process, in which the identity of a given choice in a sequence depends predominantly on the most recent previous choice outcome. Indeed, the distribution of bout lengths agreed with a Markov process (Fig. 1g), and previous reward collection patterns did not significantly influence subsequent choice timing and bout lengths (Extended Data Fig. 1e,f).

Although these data are inconsistent with a deterministic model, relative magnitudes of needs could still probabilistically influence choice. To examine this, we operationally defined thirst and hunger at any moment of a trial as the cumulative future food and water rewards that an animal would collect until satiation, and constructed a measure of normalized relative thirst and hunger ranging from –1 to +1 (Extended Data Fig. 1d). Considering each trial independently, the probability of choosing water on a given trial correlated with the mouse’s relative need (Fig. 1h). However, the most recent previous choice was significantly more predictive of current choice than need magnitudes (Fig. 1i). We next measured the probability of repeat choices across relative need values. Although the repeat choice probability decreased as the relative level of the opposing need increased, it remained generally above 80% (Fig. 1j). For trials with approximately balanced needs (relative need values between –0.25 and +0.25), choice outcomes recurred with greater than 90% probability (Fig. 1k).

These results suggest that transitions between persistent choices occur probabilistically, rather than being determined on a moment-to-moment basis by the exact balance of needs. To directly test this persistence and stochasticity, we performed transient optogenetic stimulation of channelrhodopsin-expressing RXFP1^+^ neurons in the subfornical organ (Fig. 1l) in either sated (Fig. 1m) or hungry-only mice (Fig. 1n); these RXFP1^+^ neurons (hereafter referred to as osmotic thirst neurons) are activated by increased osmolarity and their optogenetic activation produces an artificial thirst that drives drinking behaviour^24^. Sated mice that were unresponsive to Go odours transiently transitioned to choosing water upon thirst stimulation in a probabilistic manner (Fig. 1m). Thirst stimulation also promoted hungry mice to transition from choosing food rewards to choosing water rewards (Fig. 1n and Extended Data Fig. 1g), but these transitions appeared stochastic in any given stimulation epoch (Fig. 1o) and were not influenced by reward collection prior to stimulation (Extended Data Fig. 1h). In both cases, water choices persisted for at least 10 s after the termination of optogenetic stimulation (Fig. 1m,n and Extended Data Fig. 1g), suggesting the induction of a behavioural state that is partially uncoupled from the immediate optogenetic stimulation period.

In summary, in Buridan’s assay, mice autonomously organized their reward collection into persistent choice states whose sudden transitions occurred probabilistically and were modulated by relative needs. This behavioural strategy is not used only by head-restrained mice, as food- and water-restricted mice in a freely moving setting exhibited similar persistent food- or water-collection bouts with stochastic transitions (Extended Data Fig. 1i–k). Optogenetic activation of osmotic thirst neurons in head-restrained mice supported an underlying stochasticity in the behavioural response of animals to changing levels of need.

## Large-scale recording during behaviour

**Fig. 2:**
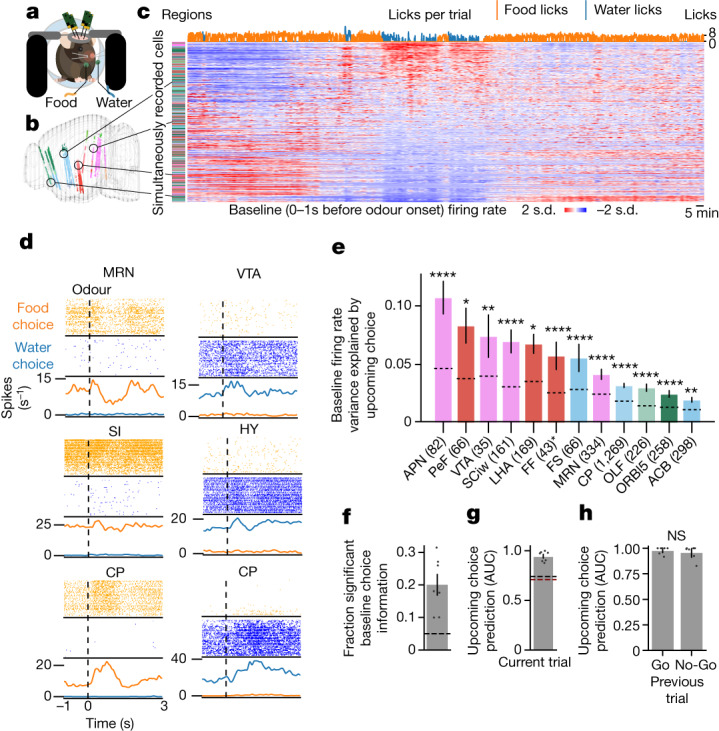
Single-unit and population neural correlates of upcoming behavioural choice. **a**, Schematic of simultaneous recording from 1,536 channels across four acute Neuropixels 1.0 probes during Buridan’s assay. **b**, Locations of neurons in the Allen Brain Atlas space. Units are colour-coded by brain region (Extended Data Table 1). **c**, An example recording session showing per-trial baseline activity for each of 996 simultaneously recorded units, *z*-scored with brain regions colour-coded as in **b**. Neurons are sorted by their correlation coefficient to the upcoming behavioural choice (top row, cumulative food or water licks per trial). **d**, Per-trial spike rasters from six example neurons (brain regions indicated on top), with spiking (ticks) shown for the first 50 food and water choices within a single session. Dashed lines indicate odour onset. Bottom, firing rate per trial. CP, caudoputamen; HY, hypothalamus; MRN, midbrain reticular nucleus; SI, substantia innominata; VTA, ventral tegmental area. **e**, Firing rate variance explained by upcoming choice, averaged within brain region. Dashed lines, null distribution per region. Exact *P* values in Methods. Bars indicate 95% confidence interval across cells. Data are pooled across recording sessions. Numbers in parentheses are counts of recorded cells in given regions; asterisks indicate regions present in only a single session. See Extended Data Table 1 for numbers of cells, mice and sessions per region. ACB, nucleus accumbens; APN, anterior pretectal nucleus; FF, fields of Forel; FS, fundus of striatum; LHA, lateral hypothalamic area; OLF, olfactory areas; ORBl5, orbital area, lateral part, layer 5; PeF, perifornical nucleus; SCiw, superior colliculus, motor related, intermediate white layer. **f**, Fraction of simultaneously recorded neurons per session whose baseline firing rates are significantly associated with upcoming reward choice, compared to a circularly permuted null (dashed line). **g**, Predictiveness of upcoming choice for held-out trials flanking switches, using population activity of simultaneously recorded neurons in the 1 s before odour onset. Dashed lines, null (circular permutation, black; session permutation, red). **h**, Population predictiveness of upcoming choice as in **g**, following either rewarded Go or unrewarded No-Go trials. Two-sided paired *t*-test, *t* = −1.072, *P* = 0.325. Mean across sessions, error bars indicate 95% confidence interval; *n* = 7 mice, 7 sessions (**f**–**h**). NS, not significant.

**Extended Data Fig. 2:**
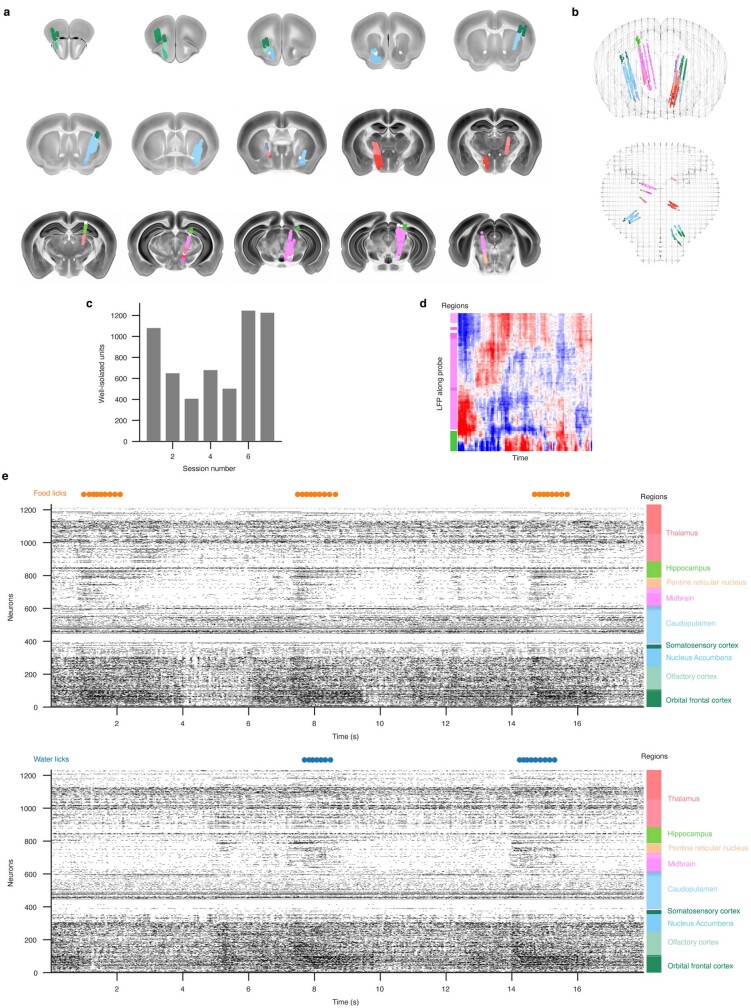
Unit anatomy and additional data in Neuropixels-based extracellular electrophysiological recordings. **a**, All well-isolated single unit locations plotted onto nearby coronal sections of the Allen Institute CCF3 2017 reference atlas. Units are color coded by the identity of the region they were assigned to according to the Allen Atlas reference colormap (Extended Data Table 1; see also panel **e**, right). **b**, Well-isolated unit locations color-coded by region assignment and plotted onto coronal (top) and horizontal (bottom) atlas projections. **c**, Counts of well-isolated units (passing quality control thresholds) that were simultaneously recorded per session. Each session is collected from a different mouse. **d**, Depth of electrodes along the insertion axis of an example probe mapped to region (color bars on the left) and aligned to local field potential (LFP) data in a 400-ms time window. **e**, Spiking activity from a single session over two 12-s time windows. Panel tops, licking for food (orange) and water (blue). Colorbars, region assignments aligned to individual neurons. Regions and region labels are color coded according to the Allen Brain Atlas colormap.

**Extended Data Fig. 3:**
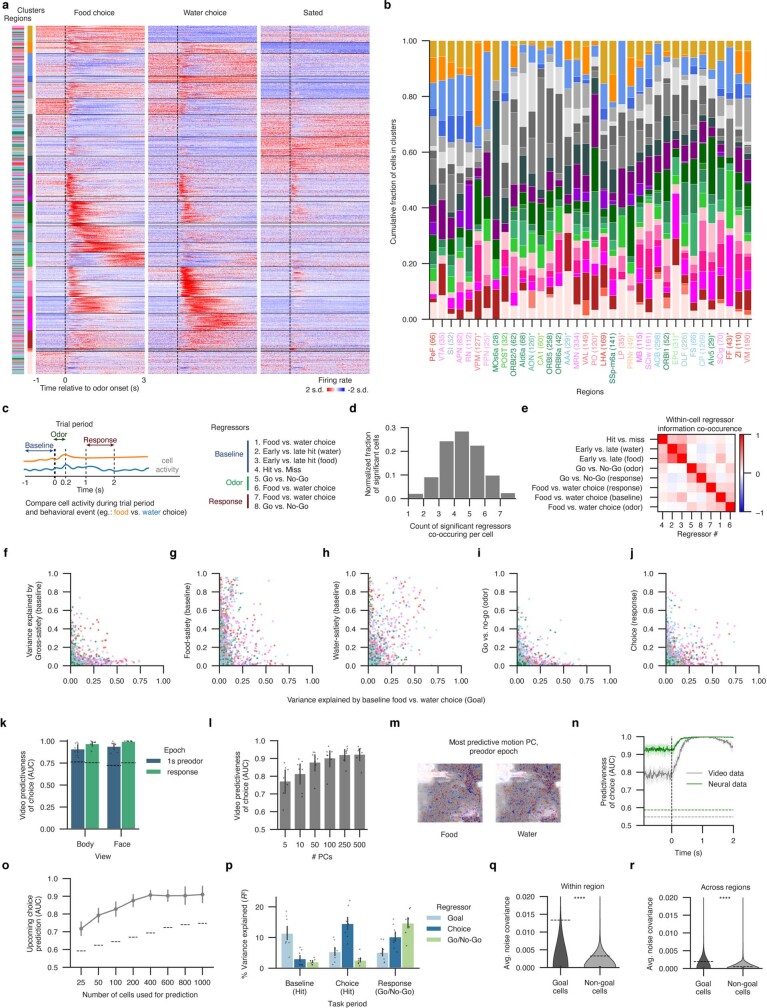
Further analyses of single neuron and neuronal population activity. **a**, Unsupervised agglomerative clustering of task- or satiety-modulated neuronal dynamics surrounding Go trials. Clusters are manually color-coded approximating the predominant information in the cluster: orange, food choice; blue, water choice; gray, satiety states; purple, odor; green, food-related action activity; red, water-related action activity. Clusters are shown separated by horizontal lines. Neurons are individually trial-averaged by condition (Go-odor food choices, Go-odor water choices, Go-odor non-responsive/sated) and Z-scored across condition. Vertical dashed line, Go-odor onset. s.d., standard deviation. Leftmost column, brain region assignments for each neuron, color coded according to the Allen Brain Atlas colormap (see Extended Data Table 1). **b**, Single-cell cluster distribution by brain region, with clusters (color coded as in **a**) plotted as a normalized fraction of cells in a given region (parenthesis: total cell numbers per region). Regions are ordered by hierarchical clustering of the regional cluster distributions. **c**, Schematic of regression analysis for functional properties of individual neurons related to task or satiety. **d**, Quantification of mixed selectivity per neuron with significant information about upcoming choice in baseline activity. The histogram shows the distribution of counts of additionally significant regressors (described in **c**) per cell. A cell is counted as significant for a given regressor if the firing rate variance explained by the regressor is greater than that of a per-cell circularly permuted null distribution, with threshold *P* = 0.05; per-cell *P*-values are not adjusted for multiple comparisons. **e**, Co-occurrence of regressors for all task- or satiety-modulated cells is shown as the pairwise correlation coefficient of variance explained by each regressor, sorted for visualization by unsupervised hierarchical clustering. The three clusters group regressors for cross-session satiety (regressors 4, 2, 3), Go vs. No-Go trial type (regressors 5, 8), and reward choice (regressors 7, 1, 6). **f**–**j**, Per-cell visualization of pairwise association between goal-information (x axes) and other regressors (y axes). Cells are color-coded according to their assigned brain region, following the Allen Brain Atlas colormap. Information at baseline about upcoming choice tended to co-occur with all regressors except Go vs. No-Go regressors. Data in **a–j** pooled from *n* = 7 mice, 7 sessions. **k–n**, Video decoding analyses. Decoding analysis from video motion data of upcoming choice. Videos of the animal’s face or body were collected at 100 Hz and the principal components (PCs) of the video motion energy were used for prediction of behavioral choice (Methods). **k**, Predictiveness (AUC) of choice by video data of the body or face (x-axis) during the 1 s pre-odor (blue bars) or the response period (1–2 s post-odor onset, green bars). Mean ± 95% confidence interval. Dashed lines, session permuted null distribution. Test trials are taken from trials flanking behavioral switches (4 trials before the penultimate trial prior to each switch, and 4 trials following the 1^st^ trial after a switch) to remove any spurious contribution of slow timescale motion covariates with choice. **l**, Face video predictiveness (AUC, y-axis) of upcoming choice during the 1 s pre-odor epoch, with variable numbers of motion principal components (PCs) used for prediction. Mean ± 95% confidence interval. **k, l**, *n* = 7 mice, 7 sessions. **m**, Visualization of the most predictive PC for food and water choices, superimposed on the average image of the animal. Pixels with high positive weights in the PC are colored red and pixels with high negative weights are colored blue. The pattern of PC weights does not indicate any obvious distinguishing motion feature of the animal, suggesting that the predictiveness of the video motion comes from more subtle “tells” of the animal’s facial motion. **n**, Predictiveness (AUC, y-axis) of the face video motion PCs (gray) or neural data (green) as a function of time, with decoders trained on each 10-ms time bin across a trial. Mean ± 95% confidence interval (*n* = 7 mice, 7 sessions). Dashed horizontal lines, circular permutation null distributions for bins in the 1 s pre-odor period. Vertical dashed line, odor onset. In summary, we can predict behavioral choice from pre-odor videos of the animal (indeed, we expect that neural activity should be reflected by animal behavior). However, predictiveness does not come from one or a few dominant motions, as the decoder requires dozens of PCs at least. Moreover, pre-odor prediction from videos of the animal’s pose is not as good as prediction from neural population activity. **o**, Analysis of redundancy of information for upcoming choice across simultaneously recorded cells. A linear decoder is trained to predict upcoming choice from the 1 s of simultaneously recorded population neural activity preceding food or water choices. The size of the population used for decoding is randomly subsampled to examine the effect on decoder performance of increasing numbers of simultaneously recorded cells, agnostic to brain region. Decoding performance (receiver operator characteristic area under the curve, AUC) is assessed for test trials flanking behavioral transitions, such that test trials with different classes occur close to each other in time and the contribution of spurious long-timescale correlations are largely removed. Mean ± 95% confidence interval (*n* = 7 mice, 7 sessions). Dashed lines, circularly permuted null distribution for each decoder. **p**, Percentage simultaneously recorded population variance explained by coding dimensions across task period. Baseline period, 1 s pre-odor activity preceding food or water choices (hit trials). Choice period, 1 s to 2 s post-odor during hit trials. Response period, 1 s to 2 s post-odor for all trials (including No-Go trials). Coding dimensions are calculated as the variance-normalized average firing rate difference between periods in trials corresponding to a given regressor (Methods). Goal regressor, 1 s pre-odor period preceding water vs. food choices. Choice regressor, 1 s to 2 s post-odor for water vs. food choices. Response regressor, 1 s to 2 s post-odor for Go vs. No-Go trials. Mean ± 95% confidence interval across recording sessions. Variance explained by each coding dimension is consistent across mice/sessions (*n* = 7) and largely distinct across regressors and their corresponding task periods. The amount of baseline period population variance explained by the goal regressor is comparable to the response period variance explained by the choice and response regressors. **q**, **r**, Cells significant for goal information have significantly higher noise covariance (covariance between firing rates in the 1 s pre-odor, with the average activity before food or water trials removed from the trial-by-trial firing rates, and evaluated at a 10-ms temporal resolution) than non-goal significant cells, suggesting that goal cells may share a common source of fluctuations or tend to influence each other’s activity more frequently than non-goal cells. Comparison for cells within the same region, **q**, and across regions, **r**. ****, two-sided t-test, *P* ≤ 1 × 10^−6^ (**q**, *t* = 11.52, *P* = 1.11 × 10^−30^, *n* = 7 mice, 21,496 pairwise correlations; **r**, *t* = 52.08, *P* = 0.0, *n* = 7 mice, 2,720,269 pairwise correlations). Y-axis is truncated. Dashed lines, means.

We next sought to explore neural mechanisms underlying the observed persistence and stochasticity in choice behaviour of mice facing conflicting needs. Previous findings have suggested that the sensory neurons underlying thirst and hunger are embedded in recurrent networks that project throughout the brain^5–8,14^. We therefore performed simultaneous extracellular electrophysiological recordings during Buridan’s assay with four Neuropixels 1.0 probes^25^ placed acutely along distinct trajectories spanning the frontal and motor cortices, basal ganglia, thalamus, hypothalamus and midbrain motor regions (Fig. 2a,b, Extended Data Fig. 2a,b and Extended Data Table 1). This strategy enabled us to synchronously record from 1,536 distinct channels, resulting in many hundreds of well-isolated units per recording session with anatomical locations recovered post hoc by atlas alignment^16^ (Extended Data Fig. 2a–d). Visualization of aligned spiking activities from all simultaneously recorded neurons suggested coordinated changes in spike rates spanning many regions, both during and between task engagement (Extended Data Fig. 2e). Unbiased clustering of trial-averaged neural activity revealed diverse functional properties, including both persistent and phasic differences between choice outcomes (Extended Data Fig. 3a). Whereas the activity of neurons in certain clusters correlated with a specific phase of the trial (for example, odour or action), other clusters were dominated by state-like neurons with persistent (throughout each trial, including before odour onset) firing rate differences between choices (Extended Data Fig. 3a). Neurons belonging to most functional clusters, including the state-like clusters, were widely distributed across brain regions (Extended Data Fig. 3b).

## Neural activity predicts upcoming choice

**Extended Data Fig. 4:**
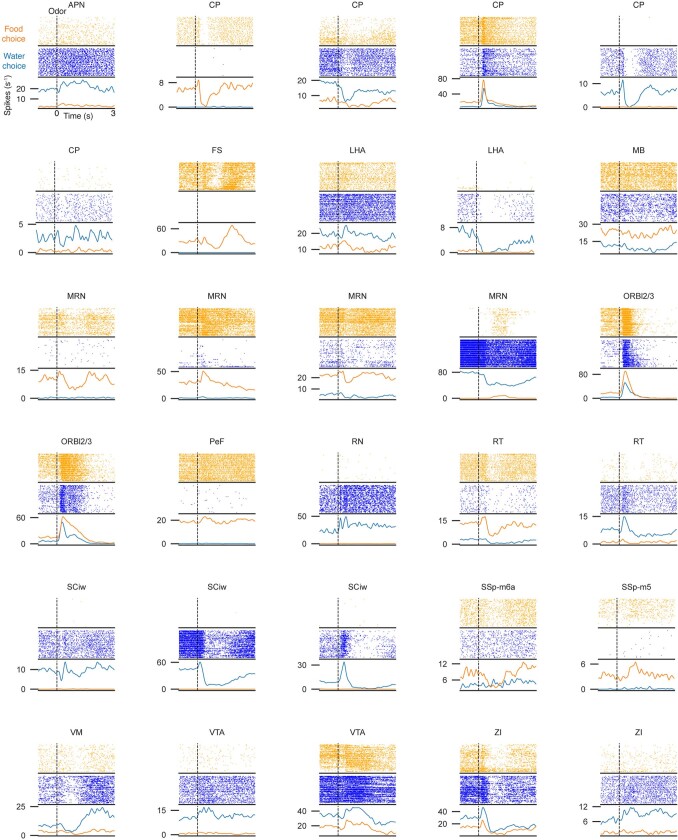
Firing patterns of example single neurons whose baseline activity correlates with upcoming behavioral choice. Example single unit spike rasters and peristimulus spike timing histograms (PSTHs). Spiking is plotted over the first 50 food-choice trials (orange, top) and the first 50 water-choice trials (blue, middle). PSTHs for food trials (orange line) and water trials (blue line) are shown at the bottom. Vertical dashed line, odor onset. Each unit is labeled by the region acronym (Extended Data Table 1) to which it was assigned. Units are sorted alphabetically according to region acronym to reflect the lack of apparent spatial organization for units with specific properties.

Given the prevalence of state-like neurons, we hypothesized that the persistence of behavioural choice is related to an underlying internal brain state of the animal. To avoid confounds with behavioural execution, we analysed neural activity at baseline (the 1 s of activity before odour onset) from all simultaneously recorded neurons across the duration of a behavioural session; during this baseline period, mice did not know when the next odour would be delivered (given the variable inter-trial intervals) or whether it would be a Go or No-Go odour. Sorting neurons by their correlation with the upcoming food choice revealed systematic changes in baseline firing rates that correlated with behavioural choice or satiety states (Fig. 2c). The spike rasters of individual upcoming-choice-correlated neurons across the duration of a trial revealed persistent firing rate differences both at baseline and after odour onset in diverse brain regions; the firing rates of many neurons were additionally modulated after odour onset (Fig. 2d and Extended Data Fig. 4).

**Extended Data Fig. 5:**
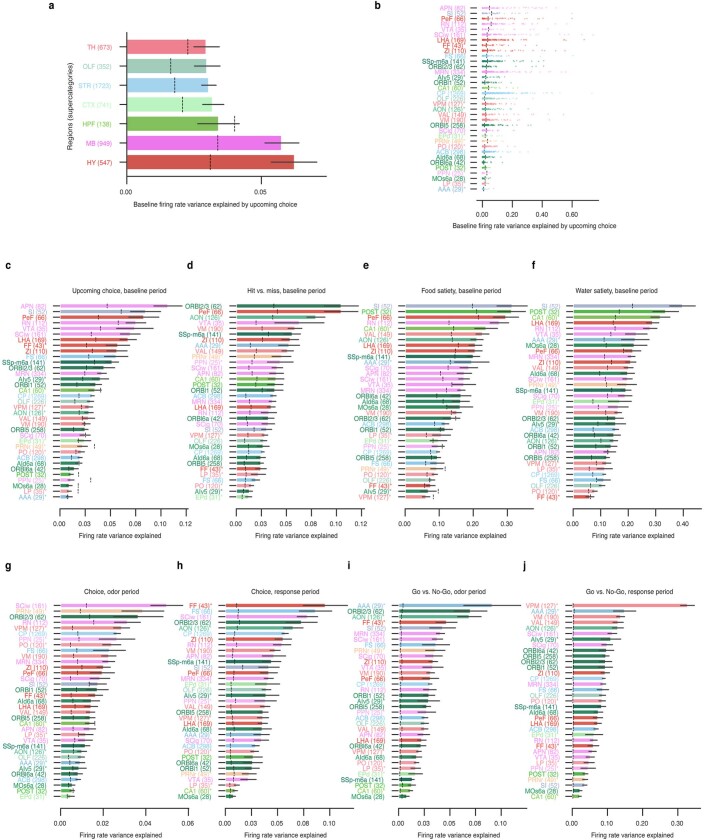
Regional distribution of regressor information. **a**, Average variance explained per-cell by upcoming choice at baseline (1 s pre-odor average) for high-level brain regions. CTXsp, cortical subplate; HB, hindbrain; OLF, olfactory areas; STR, striatum; TH, thalamus; HPF, hippocampal formation; MB, midbrain; HY, hypothalamus. **b**, Variance explained per-cell by upcoming choice at baseline; each dot represents the variance explained for a single cell in a given region. **c–j**, Average variance explained per-cell for regressors described in Extended Data Fig. 3c. For all panels, bars are averages across neurons within a given region; black lines give the 95% distribution of information per cell in a region. Numbers in parentheses are unit counts per region. Color codes are according to the Allen Institute Mouse Brain Atlas colormap. Dashed lines indicate the circular permutation null distribution for a given regressor and region. Regions are ordered by variance explained. Note that Fig. 2e shows a subset (only regions with average variance explained significantly greater than the null) of data shown in panel **c** here.

We measured how much information individual cells in each region contained in their baseline firing rates about upcoming choice using a regression analysis (Extended Data Figs. 3c and  5b). A set of hypothalamic, midbrain, striatal, and frontal cortical regions contained significantly more informative cells compared to a conservative null distribution (Methods), but with quantitative differences between regions (Fig. 2e). For example, hypothalamic and midbrain regions exhibited greater aggregate baseline firing rate information regarding upcoming choice than cortical regions (Fig. 2e and Extended Data Fig. 5a,b).

Regression analyses also revealed that most recorded neurons exhibited mixed selectivity to multiple task variables (Extended Data Fig. 3c–j), as has been previously observed in large-scale neural activity recording in different behavioural contexts^14,26–28^. Most cells with significant information about upcoming choices at baseline also contained significant information about multiple other regressors (Extended Data Fig. 3d). Pairwise analysis and unbiased hierarchical clustering of firing rate variance explained by each regressor revealed three major groupings of information mixture in cells: information related to cross-session satiety changes of the mouse (hit versus miss and early versus late), to the odour response task (Go versus No-Go), and to the choice of the mouse (food versus water) (Extended Data Fig. 3e–j).

Notably, about 20% of all recorded neurons per session contained significant information about the upcoming choice of the mouse in their baseline firing rate (Fig. 2f). The pervasiveness of this information suggested that the collective baseline activity of neurons across the brain could function as a distributed goal-like state. Indeed, we could predict upcoming choice with high accuracy using the 1-s pre-odour activity of all simultaneously recorded neurons (Fig. 2g). Whether the previous trial was rewarded or not did not significantly affect the prediction of upcoming choice (Fig. 2h), ruling out the possibility that the predictiveness of future choice was merely a reflection of previous reward. Subtle movements of the mouse were also predictive of upcoming choice (Extended Data Fig. 3k–m) and might account for some variability in the population activity^29,30^; however, neural data were significantly more predictive of upcoming choice than movement data (Extended Data Fig. 3n).

The predictiveness of upcoming choice improved as increasing numbers of simultaneously recorded neurons were included in the decoder (Extended Data Fig. 3o), and this decoding activity explained about 10% of trial-by-trial population variance in the 1-s pre-odour period (Extended Data Fig. 3p). Thus, the wide distribution of goal information across cells and regions may allow individual neurons to fluctuate on single trials because of mixed selectivity while the population together maintains state. Furthermore, consistent with a distributed goal-like network, neurons with significant goal information were more likely to be functionally coupled than cells without goal information, both within and across regions (Extended Data Fig. 3q,r). Together, these data suggest that a substantial fraction of neurons across the brain participate in a ‘goal’ state predictive of future behavioural choice. Combined with the findings of diverse phasic responses to the task and mixed selectivity, these data suggest a possible mechanism for coordination of goal information across the brain, in which fast-timescale activities unrelated to goal are superimposed on a distributed, slow-timescale, goal information carrying network.

## Forward model for the resolution of needs

**Fig. 3:**
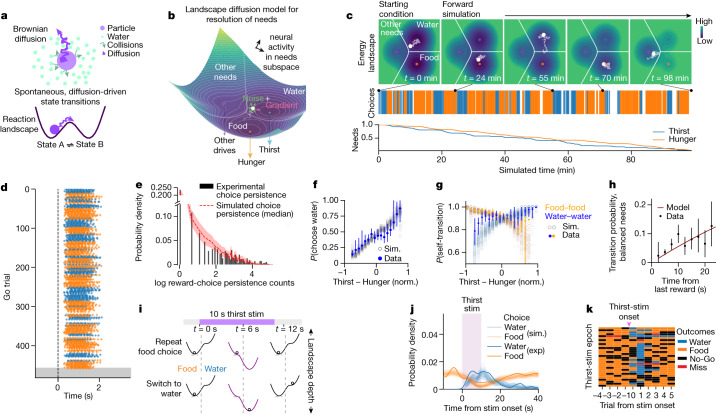
Forward model for resolving conflicting needs recapitulates behaviour. **a**, Langevin dynamics capture emergent molecular phenomena by relating observed motion to unobserved interactions via thermal noise. Top, interactions with unobserved water molecules drive a particle’s noisy Brownian diffusion. Bottom, random diffusion along an energy landscape during a reversible chemical reaction drives spontaneous transitions between reactant states. **b**, Neural landscape diffusion model for resolving conflicting needs across time. Population neural activity diffuses according to Langevin dynamics across an energy landscape whose depths are shaped by needs (arrows, bottom). Magnitudes of hunger and thirst (combining homeostatic deficits and feedforward interoceptive signals^7,8^) decrease as the animal consumes food and water, respectively. White ball, position in the neural activity subspace for needs. Landscape gradient (magenta arrow) and white noise (green arrows) jointly determine velocity. The landscape is segmented into discrete goal-related zones for distinct behavioural choices (seeking food, water or neither); position in these zones at the time of reward availability determines behaviour. **c**, Top, snapshots of forward simulation of a Buridan’s assay session. Initial thirst and hunger as well as Go and No-Go trial times are provided as inputs. Position (white dot) and the landscape (contour map; colour bar on right) evolve autonomously according to the dynamics in **b**. Squiggly line, recent trajectory. Middle, simulated trial choice outcomes shown as orange and blue lines. Bottom, recent hunger and thirst. Supplementary Video 1 shows the entire session. **d**, The simulated session in **c**, visualized with licking behaviour as in Fig. 1f. Lick timing and number are random. Dashed line, odour onset. **e**–**g**, Outcomes from 128 dataset simulations (22 sessions per dataset), analysed for and superimposed on summary statistics from 22 experimental sessions shown in Fig. 1g (**e**), Fig. 1h (**f**) and Fig. 1j (**g**). **e**, Simulated reward-choice persistence-length distribution (median ± 95% confidence interval) superimposed on experimental sessions. **f**, Simulated (sim.) probability of choosing water on rewarded trials as a function of relative need (mean) superimposed on experimental data (mean ± 95% confidence interval). **g**, Simulated probability of repeating previous reward choice (point estimates of water-to-water and food-to-food transitions) as a function of normalized thirst – hunger, superimposed on experimental data (solid dots and lines, binned self-transition probability, mean ± 95% confidence interval). **h**, Transition probability as a function of time between choices, under balanced needs. Red line, model-derived theoretical transition probability. Dots and vertical lines, experimental binned transition probability (mean ± 95% confidence interval, *R*^2^ = 0.411 for model and experiment; *n* = 15 mice, 22 sessions). **i**–**k**, Model simulation of optogenetic experiment. **i**, In a hungry-only state (high initial hunger, low thirst), optogenetic thirst stimulation is simulated by transiently deepening the thirst well. Top, example of no choice transition despite stimulation; bottom, example of transition to water and subsequent persistence after stimulation. **j**, Simulated and experimental probability densities of food choices relative to stimulation onset (purple bar). Light lines show results from each of 128 simulated optogenetic experiment datasets. Dark lines are the average experimental results (Fig. 1n). **k**, Trial outcomes surrounding each stimulation epoch (*n* = 30) from a simulated session.

**Extended Data Fig. 6:**
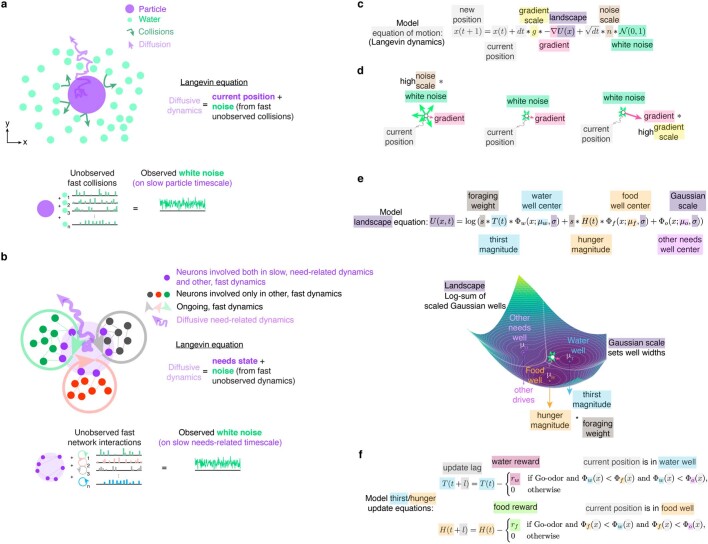
Conceptual similarities between molecular Langevin dynamics and intermixed neural networks. **a**, The Langevin equation describes the dynamics of a diffusing particle in water by transforming the unobserved, deterministic, Newtonian dynamics of water molecule collisions (green arrows) with the particle (purple) into a noise contribution to the particle’s position (bottom). This results in a stochastic description of the Brownian motion of the particle (purple arrow). A key feature of this formalism in the study of complex phenomena is the separation of unobserved fast-timescale dynamics (for example, the motion of water molecules) from the observed slow-timescale dynamics of the much larger particle. **b**, Conceptual application of the Langevin formalism to intermixed neural networks with fast and slow dynamics. Neurons involved in a distributed network with slow-timescale (for example, need-related) dynamics (purple dots) are embedded via mixed functional selectivity into additional networks of neurons (black, red, green dots) with ongoing, unobserved fast-timescale dynamics (gray, pink, light green cycles). By analogy to the Langevin picture of molecular dynamics, the activities of networks with disparate fast time-scale dynamics collide within individual mixed-selective need-related neurons to produce a stochastic noise influence on the slow-timescale dynamics of the need-related network of neurons (bottom), yielding observable diffusive goal dynamics. **c**, Equation of motion for the forward stimulation of the resolution of needs, following Langevin dynamics. $x(t)$, position of neural activity across the needs subspace at time $t$. $U(x)$, the energy landscape function. The new position is the current position plus a small differential contribution by the landscape gradient (scaled by the gradient scale $g$) and Gaussian white noise (scaled by the noise scale $n$). ${dt}$, the discretized time step. **d**, Graphical depiction of how the $n$ and $g$ terms contribute to the dynamics. Left, high noise scale $n$ relative to gradient scale $g$ results in noise-dominated dynamics. Middle, balanced scales and dynamics. Right, low noise scale $n$ relative to gradient scale $g$ results in dynamics dominated by the landscape shape. **e**, Equation for the shape of the time-varying needs landscape $U(x,{t})$. The landscape consists of a log-sum of scaled Gaussian wells. Gaussian wells, $\Phi (x)$, are defined as the negative of the multivariate probability density function. For simplicity, well centers ${\mu }_{w}$,${\mu }_{f}$, and ${\mu }_{o}$ corresponding to the wells for water, food, and other needs are set on an equilateral triangle, and the Gaussian scale parameter controlling the Gaussian widths are set to a common parameter $\sigma$. The water and food well depths are scaled by the time-varying thirst magnitude $T(t)$ and hunger magnitude $H(t)$, respectively, as well as by a “foraging” scale factor $s$ that linearly relates the experimental normalized need measurements to the model need magnitude values. **f**, $U(x,{t})$ changes across time through changes in thirst and hunger magnitudes. Thirst and hunger magnitudes are decremented after a fixed delay ${l}$ by factors ${r}_{w}$ and ${r}_{f}$ upon respective water or food reward collection; reward choices are determined upon Go-cue at time ${t\text{'}}$ by the location $x({t\text{'}})$ within a fixed segmentation of the need space into food, water, or other zones. See Methods for full implementation details.

We next aimed to formulate a minimal generative model, integrating our findings of behavioural state persistence, stochastic transitions, probabilistic influences of needs, a widely distributed neural population with goal-like information, and mixed functional selectivity of individual neurons. We made an informed guess (ansatz) at a set of governing equations inspired by the Langevin dynamics of molecular diffusion, which enables a formal description of slow dynamics in non-equilibrium systems by capturing the contribution of fast dynamics as noise^31,32^ (Extended Data Fig. 6a). We reasoned that Langevin dynamics may similarly arise in neural networks in which an interrelated set of neurons with slow-timescale dynamics (goal-related) are widely embedded in diverse neural networks with fast-timescale dynamics (Extended Data Fig. 6b). Notably, the noise that arises in the Langevin equation is a key driver of resulting macroscopic phenomena, such as Brownian motion or chemical state transitions across reaction energy landscapes^33^ (Fig. 3a). We thus formulated a set of stochastic differential equations in which need-related population neural activity diffuses across an energy landscape with wells scaled by thirst and hunger (Fig. 3b and Extended Data Fig. 6c–f). The state of need-related neural activity is partitioned into zones that specify contexts for specific behavioural goals, such as pursuing food, water, or other needs (Fig. 3b). As rewards are collected and a given need is quenched, the depth of the corresponding landscape well is diminished. The diffusion of neural activity across this needs landscape in time depends only on the local gradient at the present position in the landscape (influence of needs) and a white noise contribution (stochastic dynamics) (Fig. 3b and Extended Data Fig. 6c–f). This approach yielded a generative, forward mathematical model for need resolution.

**Extended Data Fig. 7:**
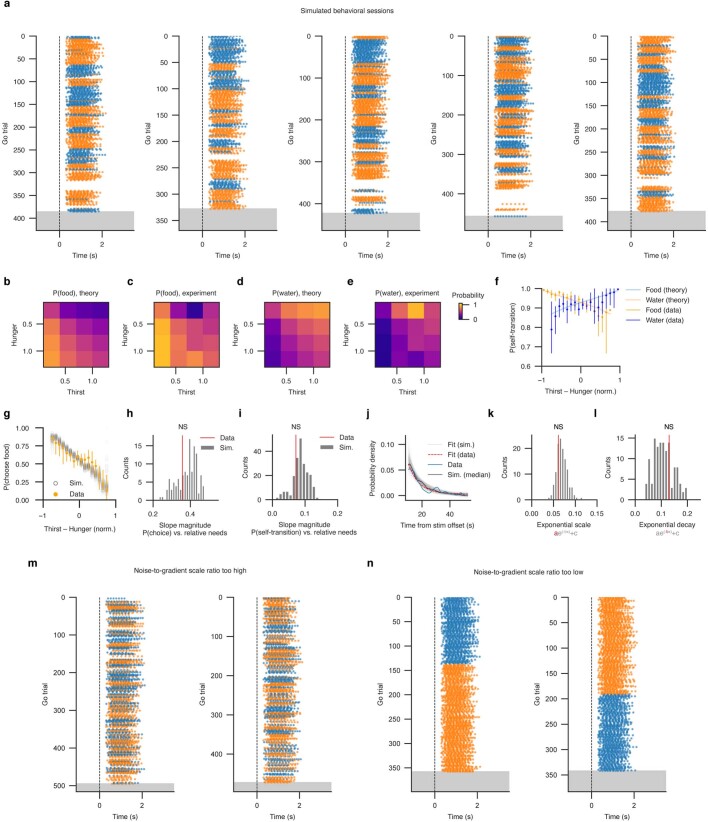
Theoretical and simulated behavioral statistics generated by the landscape diffusion model. **a**, Additional examples of behavioral simulations from the landscape diffusion model with parameters used for the analyses of Fig. 3. Simulated sessions visualized as licking behavior (orange dots, food licks; blue dots, water licks) over the course of a session until satiation (grey region) from top (session start) to bottom (session end). Lick timing and number are drawn from a uniform distribution for visualization purposes. Vertical dashed line, odor onset. **b**–**e**, Binned probability of choosing food (**b**, **c**) or water (**d**, **e**), independent of previous reward choice, as a function of Thirst and Hunger. **b**, **d**, Theoretical predictions (Boltzmann equation) derived from the model using parameters fit to trial data (Methods). **c**, **e**, Binned experimental point probability estimates. **f**, Theoretical prediction of probability of repeating previous reward choice (water-to-water, blue; food-to-food, orange) as a function of normalized Thirst – Hunger. Solid dark dots and lines, binned point estimates of the self-transition probability for normalized Thirst – Hunger values, mean ± 95% confidence interval, based on experimental data (*n* = 15 mice, 22 sessions). Light lines, model-derived theoretical prediction of self-transition probabilities, using parameters fit to trial data (Methods). The derived equations in **b**, **d**, and **f** are used in the forward model parameter fitting procedure to maximize the probability of all observed trial outcomes (Methods). **g**, Probability of choosing food on rewarded trials, independent of previous reward choice, as a function of observed relative need (normalized Thirst – Hunger; Methods), for experimental data (*n* = 15 mice, 22 sessions) and simulation (128 simulations of 22-session datasets). Solid blue dots and vertical lines, data mean ± 95% confidence interval, superimposed on open gray dots, means of simulated sessions. **h**, **i**, Quantitative agreement between simulated session statistics and experimental data. Summary statistics quantified from repeatedly performing stochastic simulations (128 times) of a 22-session dataset form a distribution of summary statistic values (gray bars), against which two-sided tail statistics can be calculated using the experimentally-observed summary statistic values (red lines). **h**, Distribution of slope magnitudes given by linear fits between the probability of reward choice and normalized relative needs. Red line, observed slope magnitude given by linear fit between experimental choice probability and normalized relative need (slope magnitude averaged between food and water choice probability fits). The experimentally-observed slope magnitude is not significantly different from the slopes computed from simulated data (gray bars), *P* = 0.664. **i**, Distribution of slope magnitudes given by linear fits between the probability of repeating the previous choice on the subsequent trial (self-transition probabilities) and the normalized relative need. Self-transition probabilities as a function of normalized relative need are linearized over the range [–1, 0] for food-to-food and [0, 1] for water-to-water, where there are greater numbers of experimental observations. Slope magnitudes for food-to-food and water-to-water are averaged together. The experimentally observed self-transition probability slope magnitude (red line) is not significantly different from those observed in repeated simulation (gray bars), tail statistic *P* = 0.734. **j**–**l**, Quantitative agreement between the simulated optogenetic experiment and the experimental dataset, focusing on the decay of water choice probability following the end of thirst stimulation in hungry animals. This statistic is not sensitive to the magnitude of thirst added during the stimulation epoch in simulations (since the landscape is assumed to have returned to its previous shape following the end of thirst stimulation) and therefore depends strongly on the diffusion dynamics. **j**, Probability density of water choices following the termination of optogenetic stimulation. Solid blue line, water choice probability in hungry animals performing Buridan’s assay based on experimental data. Solid black line, median water choice probability across repeated simulated optogenetic stimulation sessions with high hunger and low thirst. Exponential decay functions ($a{e}^{-{bx}}+c$) fit to the experimental data, red dashed line, or to each simulation of the experimental dataset, light gray lines. Each dataset with 3 sessions and 75 stimulation epochs was simulated 128 times. **k**, **l**, Distribution of exponential decay function parameters $a$ (scale, **k**), and $b$ (decay, **l**), fit to the simulated water choice probability data (gray bars) with the parameter values from experimental fits superimposed in red. Scale and decay parameters two-sided fit from experimental data are not significantly different from those fit from simulation, tail statistics *P* = 0.492 and *P* = 0.602, respectively. **m**, **n**, Additional examples of behavioral simulations from the model as above, except the ratio of the scale parameters on noise and gradient in the model has been skewed too high (**m**) or too low (**n**), resulting in disorganized behavior (**m**) or overly persistent behavior (**n**).

We simulated Buridan’s assay with our model by inputting high initial values for hunger and thirst, an initial position, and Go and No-Go trial timepoints. Running the equations forward in time produced a shifting need landscape and diffusive neural state dynamics with a resulting pattern of choices approximating that of experimental observations (Fig. 3c,d and Extended Data Fig. 7a and Supplementary Video 1; compare to Fig. 1f and Extended Data Fig. 1c). To match the experimental data, we exploited results from non-equilibrium statistical mechanics^34^ to derive from the model a set of theoretical equations for the state equilibrium and transition probabilities. Using these equations, we fit to the trial-by-trial behavioural data three fixed model parameters: scaling factors on the relative contribution of landscape gradient and noise to the dynamics, as well as a weight term on the relative scale of thirst and hunger to other needs (Methods). We used these fit parameters for the above and all subsequent behavioural simulation and analyses.

## Model recapitulates behavioural data

Theoretical equations derived from the model and fit to the trial outcome data matched the single-trial transition and choice probabilities of the data as a function of needs (Extended Data Fig. 7b–f). We then simulated each behavioural session of Buridan’s assay in our experimental dataset by matching the initial hunger and thirst magnitudes and running the generative model (Extended Data Fig. 6c) forward in time for 120 min per session. Owing to the stochastic nature of the simulation, the same initial conditions will produce distinct outputs over repeated simulation runs. Therefore, we repeated the simulation 128 times to generate distributions for all summary analyses. Analyses comparing theoretical, experimental and simulated datasets revealed both qualitative agreement and quantitative matches for key phenomena (Fig. 3e–k and Extended Data Fig. 7f–l).

Superimposition of the experimental choice persistence-length distribution onto the set of distributions in simulated sessions revealed close overlap, indicating similar underlying patterns of persistence and stochastic transitions (Fig. 3e). The distribution of choice probabilities as a function of relative need overlapped with experimental data (Fig. 3f and Extended Data Fig. 7g) and the linear slope relating choice probability to relative need was not significantly different between simulation and experiment (Extended Data Fig. 7h). Similarly, the probability in simulation of repeating previous choices was modulated by relative need in a manner that agreed with experimental data (Fig. 3g and Extended Data Fig. 7f,i). Because of the underlying diffusive process, the model predicts that without any change in need, the probability of switching choices should increase the longer an animal waits between choices. Indeed, the transition probability across increasing intervals of time between choices (using the random number of No-Go trials intermixed with Go trials) in the experimental data matched the theoretical prediction of the model (Fig. 3h).

We next simulated optogenetic activations of thirst in the context of hungry-only mice by transiently adding additional thirst in the model, with timing parameters matching those of experiment. This perturbation had the effect of temporarily deepening the energy well in the water zone (Fig. 3i). The model predicts that some stimulation epochs will result in a transition to water choices from food, whereas other epochs will have no observable behavioural change (Fig. 3i), resulting in a probabilistic effect of stimulation. Repeated simulations of the optogenetic stimulation experiment closely matched the experimental choice probabilities across the stimulation epoch (Fig. 3j); notably, transiently added thirst resulted in switches from food to water collection in some but not all epochs (Fig. 3j,k), and the decay time course of water choices back to food following the end of thirst stimulation (a phenomenon dominated by diffusion according to the model) was not significantly different between simulation and experiment (Extended Data Fig. 7j–l). Together, these results suggest that the landscape diffusion model captures the stochastic relationship between the magnitude of conflicting needs and behaviour that we observed experimentally, thus linking the contributions of state, need and noise to generate need-appropriate behaviour.

## Model predicts transition dynamics

**Fig. 4:**
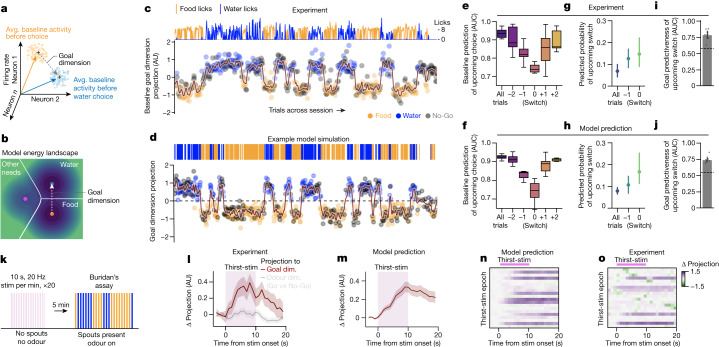
Model predicts neural state transition dynamics during natural behaviour and optogenetic thirst induction. **a**, Population activity of simultaneously recorded neurons evolves in time across an approximately 1,000-dimensional space. The goal dimension is the vector separating the average (avg.) baseline activity in the 1 s before water choices (blue arrow) from that before food choices (orange arrow). **b**, In the model, neural activity evolves in time in a two-dimensional need subspace. The goal dimension is the vector separating the energy well centre of water (blue dot) from that of food (orange dot). **c**,**d**, In both experiment and model, baseline activity dynamics across time can be measured along the goal dimension by linear projection, yielding a per-trial goal dimension activity state for water choices, food choices and No-Go trials in an experimental (**c**) or a simulated (**d**) session. The maroon line is a smoothed projection. Top, cumulative licks (**c**) or reward choices (**d**) per trial for food and water. AU, arbitrary units. **e**,**f**, Experimental results (**e**; Go trials only: *n* = 7 mice, 7 sessions) and simulation (**f**; *n* = 7 simulated sessions) of population activity predictiveness of upcoming choices surrounding trials with behavioural switches (*x* axis, trial position relative to switch trial as 0). Box plots delineate lower and upper quartiles; lines indicate median values; and whiskers span the range of values within 1.5 times the interquartile range. **g**,**h**, Predicted probability that a switch will occur on the upcoming Go trial for all trials, the Go trial before a switch (–1), or the switch trial (0) in experimental data (**g**) or simulation (**h**), using baseline population activity in the goal dimension. **i**,**j**, Goal predictiveness of an upcoming switch of the population for each session in experimental data (**i**) or as predicted by the model (**j**). Dashed line, null. **g**–**j**, Data are mean ± 95% confidence interval; *n* = 7 mice, 7 sessions. **k**, Schematic showing 20 epochs of 10-s, 20-Hz optogenetic osmotic thirst stimulation (purple bars) during Neuropixels recording without reward spout or odour. This is followed by Buridan’s assay with spout access and odour presentation. **l**, Changes in neural activity surrounding stimulation epochs are projected onto the goal dimension or the dimension separating Go versus No-Go-odour activity as a control. Values scaled by the maximum along the given dimension during subsequent behaviour. Positive values on the *y* axis are aligned with water seeking (goal dimension) and Go odours (odour dimension) during behaviour. *n* = 2 mice, 3 sessions, 61 stimulation epochs. Projection binned by 1-s intervals. Data are mean ± s.e.m. Dim., dimension. **m**, Simulation of optogenetic thirst stimulation prior to Buridan’s assay in hungry and thirsty mice. Solid line and lighter area indicate mean ± s.e.m. of change in simulating goal dimension projection over baseline (Δ projection). *n* = 3 simulations, 75 perturbation epochs. **n**,**o**, Goal activity responses to individual stimulation epochs for simulation in **m** (**n**) and for the experiment in **k** (**o**; *n* = 20 for each). Magnitudes of activity change along the goal dimension are indicated by colour codes and scaled to the peak modulation.

We next addressed how behavioural state transitions could occur if behaviour is persistent and the relative magnitude of needs does not directly drive choices. In the landscape diffusion model, transitions are emergent phenomena of the balance between landscape slope and noise-driven random walks, and thus occur spontaneously. To assess the explanatory sufficiency of the model, we sought to compare neural transition dynamics predicted by the model with those recorded experimentally. Experimentally recorded neural activity and model-simulated trajectories can be directly compared via their dynamics along a shared ‘goal dimension’ that separates upcoming water choice-related activity from upcoming food choice-related activity (Fig. 4a,b). In the recorded neural data, the ‘goal dimension’—which we define as the difference between average baseline population activity before water choices and before food choices—was extracted with a linear classifier; neural population activity along the goal dimension at a specific time was measured by linear projection (Fig. 4a and Methods). In the model, these dynamics were measured by taking the simulated position in time along the vector from the centre of the hunger well to the centre of the thirst well (vertical axis in Fig. 4b).

**Extended Data Fig. 8:**
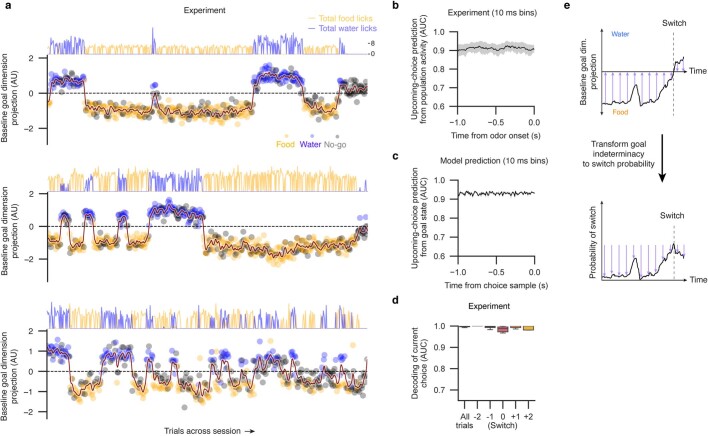
Additional experimental examples on goal activity and decoding analyses. **a**, Activity along the goal dimension in simultaneously recorded neural activity for three additional sessions. Measurements (baseline population activities projected onto the goal dimension) are shown per-trial for 0–1 s prior to water choices (blue dots), food choices (orange dots), and No-Go trials (black dots). Projection magnitude shown in arbitrary units (A.U.). Maroon line, projection smoothed by a Butterworth filter for visualization. Left, session start; right, session end. Top, corresponding cumulative licks per trial for food (orange) and water (blue). **b**, Predictiveness for upcoming choice from population neuronal activity in experiments, fit on a 10-ms resolution. Mean, solid line; ± s.e.m., gray area. *n* = 7 mice, 7 sessions. **c**, Model prediction for baseline persistence of population upcoming-choice predictiveness (AUC, receiver operator characteristic area under the curve) on a fast timescale (10 ms bins, Methods). *n* = 7 simulated sessions. **d**, Decoding performance (AUC, y-axis) of current reward from average population firing rates 1–2 s post-odor onset surrounding hit trials with behavioral switches (x-axis, trial position relative to switch-trial labeled 0). AUC values for all trials for each trial position relative to switches are averaged within session. *n* = 7 mice, 7 sessions. Boxplots span lower and upper quartiles of distributions; lines indicate median values; whiskers indicate the range of values within 1.5 times the interquartile range. Firing rates following reward choice remain highly predictive of recent reward identity surrounding behavioral switches. This contrasts with Fig. 4e, in which decoding performance of the upcoming choice from baseline activity is diminished in trials both before, during, and following behavioral switches. **e**, Schematic illustrating the transformation of population goal activity into a probability of upcoming switch. Top, schematized population activity along the goal dimension (black line) near a switch (dashed vertical line). As observed in Fig. 4e, activity along the goal dimension loses discriminability (AUC towards 0.5) for reward choices near switches. The magnitude of the distance of the goal-activity from the center line (purple arrows) is linearly rescaled into a probability of upcoming switch (bottom). Thus, upcoming switches become more predictable than chance (Fig. 4i) by transforming the degree of indeterminacy of the current population activity (regarding whether the upcoming choice will be food or water) into a probability that a switch will occur.

We compared experimental neural dynamics with the model along the goal dimension for each trial in a given behavioural session (Fig. 4c,d and Extended Data Fig. 8a). In both experiment and model, we found baseline population activity along the goal dimension to be persistent within contiguous reward-choice outcomes, including the intermingled No-Go trials, with minimal slow-timescale variation between behavioural switches. Thus, neural population activity along the goal dimension at a given point in time could function as an ‘internal goal state’ that underlies the persistent behavioural states that we observed. As predicted by the model, we observed fast-timescale noise-like variation in the experimental per-trial neural activity along the goal dimension (Fig. 4c and Extended Data Fig. 8a). Moreover, the model predicts rapid trajectories along the goal dimension during behavioural state transitions (owing to the landscape saddle between wells and pull of the landscape gradient). These rapid transition dynamics along the goal dimension were readily observable in both experimental neural activity (Fig. 4c and Extended Data Fig. 8a) and simulated trajectories (Fig. 4d).

**Extended Data Fig. 9:**
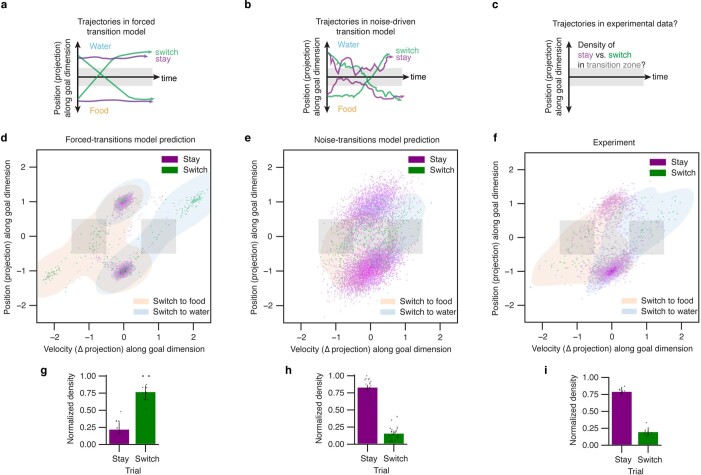
Comparison between forced and noise-driven transition models. Here, we consider whether state transitions could be explained by an alternative landscape model in which transitions are forced by external input (e.g., from an unobserved neuronal population with distinct dynamics), rather than driven by internally noisy dynamics. Near the decision boundary, trajectories of a model with forced transitions are different from trajectories of a model with noise-driven transitions; non-transition trajectories that approach the boundary should only occur in the noise-driven model but not in the forced-transition model. We quantified these trajectories in simulations of each model and in the experimental neural data. **a, b**, Schematics contrasting the behavior of goal state trajectories near the boundary of food and water zones, under a landscape model with (**a**) externally-forced transitions or (**b**) noise-driven transitions. Green lines, trajectories resulting in a switch between food and water zones; purple lines, trajectories that stay in their initial zone. Grey region, zone adjacent to the boundary. **c**, Schematic illustrating the analysis approach to differentiate models. **d–f**, Phase portraits of simulated or experimental neural data. The projection onto the goal dimension (position) is plotted on the y-axis and the trial-to-trial difference of the projection onto the goal dimension is plotted on the x-axis. The trial-by-trial difference in position along the goal dimension can be considered as the rate of change of the position across the average interval of time between trials, which we denote the velocity. Data is plotted as a joint density (2D histogram) of position and velocity values for all trials. Purple marks, density of trials with the same reward choice as the previous (stay); green marks, density of trials with a reward choice switch. Blue area, kernel density estimate (KDE) for transitions to water; orange area, KDE for transitions to food. These areas highlight the phase space in which transition dynamics occur. **d**, Simulation results from the forced-transition landscape model. Force magnitude was set to be minimally sufficient to cross the decision boundary in the average time between two trials, resulting in a state transition. Noise magnitude was set to be insufficient to generate spontaneous transitions. **e**, Simulation results from the noise-driven landscape model. **d**, **e**, 21 simulation runs for each model. **f**, Phase portrait generated from experimental neural data across all recording sessions (*n* = 7). **g–i**, Normalized densities of stay (purple) and switch (green) trials were quantified in the transition zones (gray squares in **d–f**) through which switch trajectories pass. These zones were defined as the boundary regions in the transition trial phase space, lying between the average stay-trial food and water densities (at [–0.5, +0.5] with velocity [–0.5, –1.5] for transitions to food; and at [–0.5, +0.5] with velocity [+0.5, +1.5] for transitions to water). Normalized density quantification in these regions for the forced-transition model (**g**), noise-driven transition model (**h**) and experimental data (**i**, *n* = 7 mice, 7 sessions). Mean density in the zone ± 95% confidence interval. In both analyses, experimental data (**f**, **i**) are consistent with simulations based on the noise-driven transition model (**e**, **h**) but not consistent with simulations based on the forced transition model (**d**, **g**).

Despite the noisy trial-by-trial fluctuations in fast-timescale activity along the goal dimension, both the experimental neural data and model remained highly predictive of upcoming choice in the 1 s before odour onset (Extended Data Fig. 8b,c). Although this was the case on average, the model also suggests that alternative dynamics take place before behavioural switches: the spontaneity of choice transitions with respect to behavioural trial times and the proximity of noisy transition trajectories to the decision boundary implies that activity just before a behavioural switch should lose predictiveness for upcoming choice. Indeed, this was apparent in analysis of baseline activity for trials surrounding behavioural switches, for both experimental data (Fig. 4e) and model simulations (Fig. 4f). We note that the loss of baseline predictiveness of choice just before switches also suggests that the population goal state is not merely persistently reflecting the identity of the most recent reward (Fig. 2h and Extended Data Fig. 8d). Conversely, if the population activity loses choice discriminability near switches, then a lack of choice discriminability in the neuronal population activity at any moment in time should be predictive of an upcoming switch. Indeed, for both experimental data (Fig. 4g) and model simulations (Fig. 4h), the predicted probability of an upcoming switch, based solely on the distance of activity along the goal dimension from the midpoint (Extended Data Fig. 8e), increased just before behavioural switches compared with all other trials. Furthermore, the magnitude of goal dimension activity at baseline alone could predict upcoming switches in both the experimental data (Fig. 4i) and the model simulations (Fig. 4j). We additionally found that the transition dynamics of experimental data agreed with the noise-driven transition model but not with a forced-transition model (Extended Data Fig. 9).

## Causal test of model predictions

Finally, we sought to test the causal link between thirst sensation and internal goal state dynamics as described by the model. To avoid behavioural confounds, we performed Neuropixels recordings while optogenetically stimulating osmotic thirst neurons during a quiet waiting period (stim epoch) without odour or reward spouts; this was followed by our standard Buridan’s assay in the same session (Fig. 4k). This experimental scheme enabled us to construct the goal dimension on each session from neural activity during the unperturbed Buridan’s assay, while still measuring changes in neural activity during the preceding repeated thirst perturbations along the goal dimension.

**Extended Data Fig. 10:**
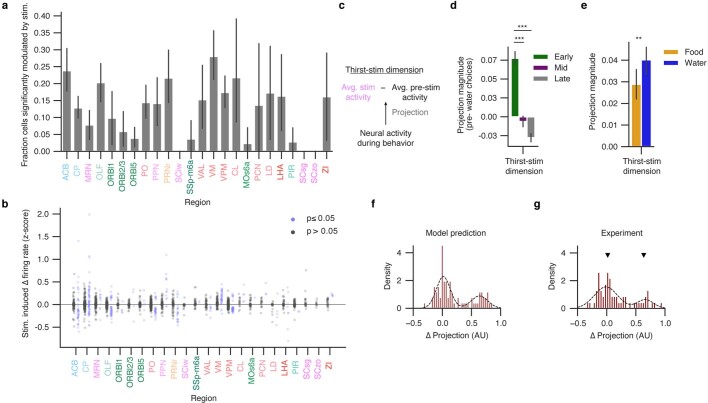
Additional analyses of optogenetic perturbations during recording. **a**, **b**, Modulation of brain regions by optogenetic stimulation of SFO Rxfp1+ osmotic thirst neurons. Neurons were defined as significant if their mean firing rates during optogenetic stimulation were different from their mean firing rates prior to stimulation, two-sided t-test with significance threshold *P* ≤ 0.05, *n* = 20 stimulation epochs per cell. **a**, Fraction of cells within each region that were significantly modulated by thirst stimulation. Mean ± 95% confidence interval. Region labels are color coded by the Allen Brain Atlas colormap. **b**, Modulation of individual neurons (dots) in each region, neurons color coded by significance of modulation across multiple stimulation epochs. Y-axis, change in firing rates (z-scored across the entire recording session). **c**, In a complementary analysis of the thirst perturbation prior to Buridan’s assay, shown in Fig. 4l–o, a thirst-stim axis (difference between average activity during and before optogenetic stimulation) is constructed and neural activity from the subsequent behavior period is projected onto it in **d** and **e**. **d**, Projection onto the thirst-stim axis of baseline population activity preceding water choices, for early rewards (first third), middle rewards (middle third), or late rewards (last third) in a session. ***, one-sided t-test; early versus mid, *t* = 21.47, *P* = 1.21 × 10^−86^; early versus late, *t* = 11.82, *P* = 3.96 × 10^−24^; *n* = 674 early trials, 511 mid trials, 103 late trials. Mean ± 95% confidence intervals. **e**, Projection onto the thirst stim axis of baseline population activity preceding food trials (orange) or water trials (blue). **, one-sided t-test, *t* = −2.33, *P* = 0.01; mean ± 95% confidence intervals. In summary (**c–e**), neural activity characteristic of thirst stimulation correlates with behavioral satiety (**d**) and increases more before water choices than before food choices (**e**). **f**, **g**, Analysis of the variability in neural responses along the goal dimension upon simulated (**f**) or experimental (**g**) optogenetic stimulation epochs, related to Fig. 4n,o. Change in activity along the goal dimension was averaged in the last two seconds of each stimulation epoch across all simulated or experimental sessions. The distribution of these changes in activity were visualized as normalized histograms. **f**, The statefulness and indirect influence of needs in the model predict a bimodal distribution (dashed lines, two Gaussians fit by a mixture model) of activity changes along the goal dimension, with many simulated stimulation epochs not resulting in significant change along the goal dimension (left Gaussian). **g**, In line with the model predictions, activity changes along the goal dimension across all experimental epochs (*n* = 61) also followed a bimodal distribution (dashed lines, Gaussians fit by mixture model to the experimental data) with many optogenetic stimulation epochs yielding no change in goal dimension activity. Arrowheads indicate the mean locations of the Gaussians fit to the simulated data. For both simulated and experimental data, model comparison (Bayes’ Information Criteria, BIC) favored a bimodal distribution over a unimodal distribution (unimodal Gaussian fit to simulated data, BIC = 47.966; bimodal Gaussian mixture fit to simulated data, BIC = 13.223; unimodal Gaussian fit to experimental data, BIC = 48.374; bimodal Gaussian fit to experimental data, BIC = 45.042).

The landscape diffusion model made several key predictions about this experiment: (1) activity along the goal dimension should move, on average, towards the water-seeking zone during optogenetic stimulation; (2) even in the absence of behaviour, changes in activity along the goal dimension should slowly decay after stimulation offset; and (3) only a subset of stimulation epochs should result in a change of activity along the goal dimension towards the water-seeking zone (Fig. 3i). We simulated the thirst stimulation experiment by initializing the model with high values for thirst and hunger and then transiently adding thirst magnitude with timing matched to the experimental stimulation parameters. We found that in both experiment (Fig. 4l) and simulation (Fig. 4m), activity moved in the direction of the water-seeking zone along the goal dimension during thirst stimulation and declined slowly from its peak following the end of stimulation. As a control for the experimental data analysis, activity did not significantly change along a similarly constructed dimension discriminating Go from No-Go odours (Fig. 4l). Cells significantly modulated by optogenetic stimulation were distributed across multiple brain regions, with quantitative differences in frequency (Extended Data Fig. 10a,b). Complementary analyses supported the causal link between thirst and internal goal state (Extended Data Fig. 10c–e). In both simulation and experiment, individual epochs of thirst stimulation exhibited stochastic dynamics as predicted by the model, with some individual goal activity trajectories appearing to transition to and persist in a goal state associated with water-seeking, whereas others exhibited no obvious change (Fig. 4n,o and Extended Data Fig. 10f,g). For the experimental neural data, this variability within an animal occurred despite the same external experimental parameters and internal homeostatic deficit states.

Collectively, these data demonstrate a causal link between increasing osmotic thirst neuron activity and moving the internal goal state towards water seeking. They lend support to the indirect effect of homeostatic deficits on behaviour, as described by the landscape diffusion model. These results further indicate that the stochastic resolution of conflicts between needs is not only a behavioural phenomenon but also a neural phenomenon that can be dissociated from overt goal-seeking motor actions.

## Discussion

**Fig. 5:**
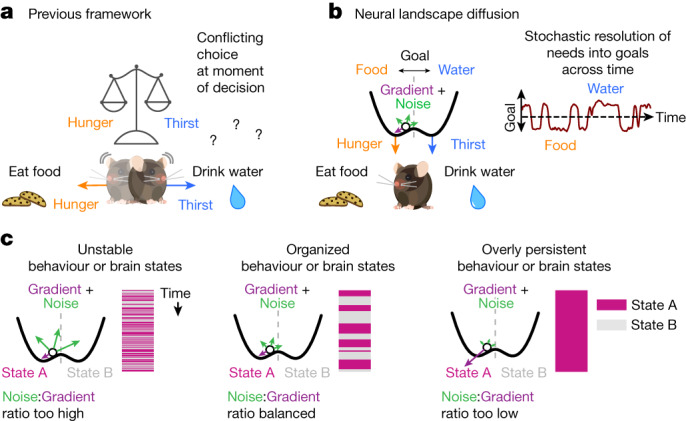
Neural landscape diffusion as a framework for the continuous organization of brain states across time. **a**, Previous framework in which needs act directly as forces on behaviour, leading to behavioural conflict under equal needs at the moment of choice. **b**, In the neural landscape diffusion model, needs act indirectly on behaviour by reshaping an underlying energy landscape. An intermediate goal state diffuses across the landscape directed by landscape gradient and noise. The goal state position at the time when choice is presented determines what decision an animal would make. Owing to the stochastic nature of diffusion, the current goal state may change randomly across time, even without changes to the underlying needs (right). **c**, Scale parameters controlling the relative strength of landscape gradient and noise can shift the behaviour of the system across regimes of varying stability. Left, excess noise relative to gradient results in unstable states with numerous transitions. Right, excess gradient relative to noise results in overly persistent states that fail to spontaneously transition and remain stuck. Middle, balanced noise and gradient generate organized, sticky behavioural states with spontaneous transitions.

Using thirst and hunger in mice, we explored the behavioural and neural dynamics of conflicting needs to reveal principles of an underlying neural control system that organizes behaviour across time. Unexpectedly, similarly hungry and thirsty mice made persistent choices to seek food or water and transitioned between choice bouts in a stochastic manner. Quantitative analyses indicate that the relative magnitude of needs modulates behavioural choices in a probabilistic manner. The persistence of behaviour despite shifting needs suggested an internal mechanism that maintains a goal state guiding upcoming choices. We found widely distributed neural correlates of this goal state in simultaneous recordings performed during behaviour, most notably the persistent baseline population activity along the goal dimension that coincides with reward-choice outcomes (Fig. 4c). Neurons that contained significant goal information also exhibited mixed selectivity for other fast-timescale features of the behaviour. We proposed a conceptual model in which goal-related neural activity diffuses across an energy landscape of needs to organize behaviour across time. Theoretical predictions and simulations from a mathematical realization of the model captured behavioural phenomena and neural dynamics with minimal free variables. Thus, rather than acting as a direct force on behaviour (Fig. 5a), our experimental data and modelling suggest that thirst and hunger indirectly drive shifts in behaviour by reshaping an underlying energy landscape and thus biasing the stochastic movements of an internal goal state (Fig. 5b).

Our data and model resolve the quandary of Buridan’s ass via a goal-like brain state whose position in neural space determines behaviour, rather than a direct comparison of relative needs. According to this framework, the donkey’s mind is made up before it is given a choice; and if the donkey is made to wait, then its choice may spontaneously switch. Even in the case where the goal state lies at a decision boundary between behavioural outcomes and the magnitudes of hunger and thirst are equal, our model and experimental results suggest that this symmetry is spontaneously broken^35^ by random fluctuations in the internal state near the saddle between energy wells.

**Extended Data Fig. 11:**
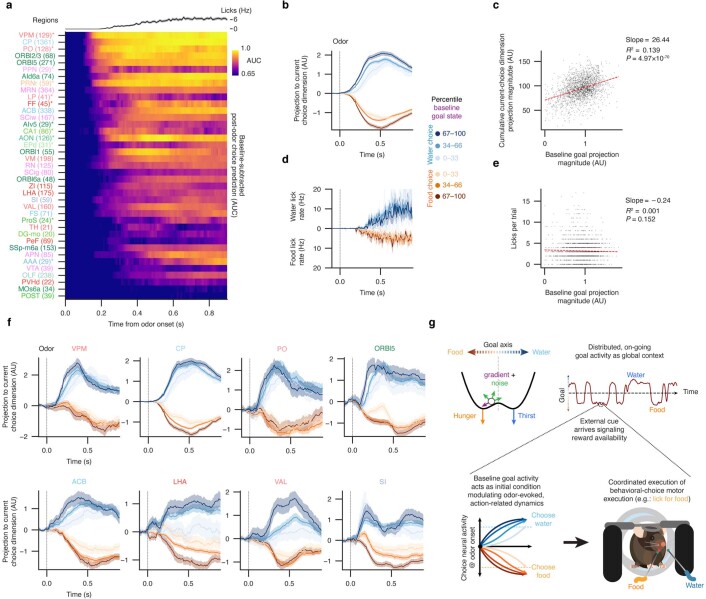
Goal-related activity at baseline influences regional choice activity after Go-odor onset. We found that persistent goal-related information is widely distributed across the brain and precedes external cues of reward availability. However, after odor onset, distinct population activity patterns arise that further differentiate Go vs. No-Go odors and choices of food vs. water (Extended Data Fig. 3). Here, we explore the relationship between distributed goal-related neural activity and regional odor-evoked activity dynamics by 1) examining activity that distinguishes food vs. water choices after Go-odor onset (we term hereafter as “current choice activity”) across different brain regions and 2) interrogating whether current choice activity might be influenced by the baseline goal-related activity prior to odor onset. **a**, Performance of *baseline-subtracted* regional population activity for predicting choice after Go-odor onset, evaluated per time-bin (AUC). Brain regions are sorted according to the first time-bin in which AUC reached half-max (0.75). Top, average lick rate across all trials and sessions post-odor onset. Mean, solid line; ± 95% confidence interval, shaded area. Region labels are color-coded according to the Allen Brain Atlas regional taxonomy and colormap (Extended Data Table 1). Note that we used baseline-subtracted regional population activity to predict choice after Go-odor onset in order to remove the influence caused by the fact that baseline population activities in different regions contain different amount of information about upcoming choice (Fig. 2e). The earliest regions gained increased predictiveness over baseline at least 100 ms before the average onset of licking; these regions could play a functional role in finalizing behavioral choices or in initiating choice-specific motor plans. Some of the earliest regions, including the orbital frontal cortex (OFC) and caudoputamen (CP), have been previously described as playing roles in value-guided decision making^71–73^. Cell numbers are in parentheses. Regions with fewer than 15 cells were excluded from the analysis. **b**, Because individual cells have mixed selectivity between goal-, odor-, and action-related factors (Extended Data Fig. 3), we hypothesized that population variation along the goal axis at baseline (pre-odor) could predict variation in baseline-subtracted current choice activity dynamics. We therefore analyzed within choice variation in the projection of baseline-subtracted population neural activity onto a dimension that separates food from water choices in the response period (1–2 s post-odor), as a function of the magnitude of the population projection along the goal axis (1 s pre-odor epoch, predictive of upcoming choice). Projections for water choices (blue lines) and food choices (orange lines) are binned into percentile subsets based on per-trial baseline variation along the goal dimension. Bin mean, solid line; ± 95% confidence interval, shaded area. Vertical dashed line, odor onset. **c**, Per-reward-trial relationship between condition mean-subtracted, baseline goal-related activity and baseline-subtracted current choice activity magnitudes. Baseline-subtracted activity projected along the current choice dimension is summed from 100 ms – 900 ms after odor onset (y-axis). Dots, individual trials. Dashed red line, linear fit, indicating a significant correlation between fluctuations along the goal dimension at baseline and subsequent choice dynamics. **d**, **e**, Unlike the current choice activity, within-choice variation in baseline goal activity had no effect on the timing or intensity of licking towards food or water. This suggests that, while variation in the baseline goal-activity within choices may alter odor-evoked neural dynamics in the brain, the behavior that ultimately results is categorical and has consistent timing regardless of variation in population goal activity. n = 7 mice, 7 sessions (**b**–**e**). Since different brain regions have different upcoming and current choice activity time-courses and dynamics (**a**), we next analyzed whether individual regions might exhibit distinct patterns of modulation by baseline goal activity. **f**, Projections of baseline-subtracted neural activity onto the current choice dimension following odor onset (vertical dashed line) for 8 selected regions, analyzed and visualized as in (**b**). Top row, example regions whose baseline-subtracted current choice activity has faster onset dynamics for either food or water choices with high baseline goal activity magnitude vs. trials with low baseline goal activity magnitude. Notably, these regions had some of the earliest onset of choice predictiveness beyond baseline (**a**). Bottom row, example regions whose baseline-subtracted current choice activity is modulated by baseline goal activity late in the response period. Regions with fewer than 10 recorded cells per session were excluded from the analysis. The different time courses of modulation of regional current choice activities suggest that baseline goal-related information may influence distinct brain functions related to choice—for instance, combining goal state with information about reward availability to finalize response selection (occurring rapidly after odor onset), or adding goal context to execution and reward-related activities (occurring during licking and reward collection). **g**, In summary, ongoing baseline goal-related population activity (top) may function as an initial condition that shapes odor-evoked dynamics (bottom left), coordinating regional dynamics such that appropriate behaviors emerge (bottom right) despite conflicting needs. For all regional analyses (**a**, **f**), see Extended Data Table 1 for number of animals and sessions used.

We next consider how the global goal-like context influences subsequent behavioural choice. Prior work has suggested that interconnected groups of neurons may implement actions via shared dynamics^36–39^. In this conceptual framework, sensory inputs^37,38^, inter-regional communications, neuromodulatory tone^40^, or other features of internal state^14^ may create initial conditions that result in distinct behavioural outcomes. Indeed, we observed that baseline goal-related activity influences regional choice activity after odour onset (Extended Data Fig. 11), suggesting that the broadly distributed goal state activity could function as a shared initial condition to coordinate the population neural dynamics of distinct regions in the production of specific behavioural outcomes. In this way, goal-related activity in a large fraction of neurons could have no direct effect on action at baseline, while nonetheless specifying neural dynamics^41^ that generate action following the odour cue. The separation of longer-term plans from the implementation of behavioural actions enables more hierarchical motor planning, more robust learning and simplified reward assignment^42^.

Our recordings did not reveal the primacy of any one region in controlling transitions between choices given balanced needs. Although we recorded activity from many regions, our sampling included only a small fraction of the brain, and it remains possible that our recordings missed key effectors or modulators of transitions. Nonetheless, we note that the Langevin-like model we propose here explains both natural and optogenetically induced transitions that agree with experimentally observed statistics without requiring any transition controller input. Moreover, computational analysis of the goal state dynamics was inconsistent with an external driver of transitions (Extended Data Fig. 9).

Key properties of our proposed model include: (1) the remodelling of the underlying energy landscape; (2) maintenance and update of position in the need subspace; and (3) scaling terms for both the landscape gradient and noise. The remodelling of the energy landscape could be physically realized by the broad release of state-related neuromodulators^43,44^, by synaptic reweighting^45^, or by other network activity mechanisms^46^. Identifying the neurobiological mechanisms tuning the gradient and noise scaling factors may be an important aim for future studies. The balance between these scale factors determines the rate of transition in the model: a high noise scale factor leads to frequent transitions with short dwell times, and a high gradient scale factor keeps subspace activity stuck in one well (Fig. 5c and Extended Data Fig. 7m,n).

The qualities of persistence and sudden transitions in internal state that we found in our assay share important features with the adaptive and maladaptive transitions of emotional and psychological states in humans. Intriguingly, major morbidity in schizophrenia arises from disorganized thought processes and behaviours, characterized by the abnormal persistence of, and transitions between, cognitive and behavioural states^47,48^. These debilitating symptoms lead to disruption of daily life activities including self-care, eating and drinking, as well as unstable emotional states and thought processes; this behavioural disorganization in time is evocative of an excess in the noise term of our model (Fig. 5c, left). On the other extreme, certain maladaptive conditions could arise from minimizing this noise term (Fig. 5c, right); for example, reduced ease of brain-state shifting could contribute to stereotyped and restricted behavioural patterns for those on the autism spectrum, and to behavioural symptoms in other disorders characterized by reduced exploration of available action space (such as major depression). Future work will elucidate to what extent our results generalize to diverse homeostatic needs and affective states in mice and in humans, and whether the model we describe may ultimately help to frame our understanding and treatment of psychiatric diseases.

## Methods

### Experimental model and subject details

Female wild-type (*C57BL6/J*, Jax 000664) or *Rxfp1*^*em1(cre)Ngai*^ (*Rxfp1-P2A-cre*, a gift from J. Ngai) mice were used for experiments. Experimental procedures were conducted on mice beginning at age 6–12 weeks. All animal procedures were conducted following guidelines approved by Stanford University’s Administrative Panel on Laboratory Animal Care (APLAC) and guidelines of the National Institutes of Health.

### Surgical procedures

Sterile techniques were used throughout the duration of surgical procedures. Mice were anaesthetized with 1–2% isofluorane and given sustained release buprenorphine (0.5 mg kg^−1^) prior to surgery. Following stereotaxic affixation, the head was cleaned with betadine antiseptic solution (Betadine) and 70% isopropanol wipes. The scalp and periosteum were removed and the skull cleaned thoroughly with 3% hydrogen peroxide solution and saline. Once the skull had dried completely and was level, a custom stainless steel headbar was affixed over the cerebellum with clear dental cement, and a thin layer of clear dental cement was applied to the surface of the skull, forming a bowl with the headbar. The position of bregma was marked for later reference. In the case of subjects used for optogenetic experiments, *Rxfp1-P2A-cre* mice were prepared as described above. Additionally, AAV5-Ef1a-DIO-hChR2(H134R)-eYFP^49^ (350 nl of 5 × 10^12^ viral genomes per ml titre) was injected into the subfornical organ (SFO, –0.65 A/P, 0 M/L, –2.75 D/V relative to bregma; unit for all stereotactic coordinates is mm) at 100 nl min^−1^ using a Hamilton syringe. The injection bolus was allowed 10 min for diffusion prior to withdrawing the syringe. Following the injection, a 400-µm fibre optic with a 1.25-mm cannula was implanted at a 30° angle from the dorsal–ventral axis above the SFO (–0.65 A/P, +1.4 M/L, –2.76 D/V) and affixed to the skull with dental cement.

One day prior to Neuropixels recordings, mice were anaesthetized with isofluorane as described above and craniotomies were drilled in 4 locations on the skull: frontal cortex: 2.25–2.5 A/P, 1.5 M/L; dorsal striatum: 0.3–0.5 A/P, 3.15 M/L; hypothalamus: 2.2 A/P, 2.2 M/L; midbrain: 3.05–3.3 A/P, 1.5 M/L. The Neuropixels insertion trajectories were initially chosen to sample regions previously reported to be involved in stimulus-value association (frontal cortex), homeostatic needs and consumption (hypothalamus, especially lateral hypothalamus), action selection and behaviour initiation (striatum), and motor execution and reward (midbrain). Given these regions, we refined coordinates based on long-range axon projection data between regions (Allen Brain Institute anterograde projection dataset^50^) to maximize our chances of recording simultaneously from multiple interconnected nodes of a circuit. Craniotomies were cleaned with saline and covered with Kwik-Cast (World Precision Instruments) until recordings. A reference electrode (platinum-iridium wire, 0.002-mm diameter, A-M Systems) was inserted over visual cortex and affixed with dental cement.

### Behavioural training for Buridan’s assay

Mice were allowed at least one week to recover following surgical procedures. Mice were maintained on a reverse light–dark schedule and experiments were performed in dark periods or early light periods. Mice were placed on a food and water restriction schedule approximately 1 week prior to behavioural training. Mice received approximately 3 g of dry food and 1 ml of water at the same time each day, with amounts adjusted to maintain mice above 80% baseline body weight. Once mice were reliably performing behavioural tasks, daily water allotment was obtained during behavioural sessions, and additional dry food (0.5–2 g) was supplemented depending on body weight and the amount of food collected during a behavioural session.

Mice were trained on a custom behavioural rig consisting of a two-odour olfactometer, a head-fixation apparatus, two reward delivery spouts—one delivering salted (0.5 M NaCl final concentration) liquid vanilla Ensure (Abbott), the other delivering drinking water—and a high speed (200 fps) colour camera (Basler Ace acA1300-200uc USB3) used for tongue detection (custom detection code, implemented using BonsaiRx^51^, with a measured detection latency of 5–10 ms (1–2 camera frames)). Behavioural protocols were controlled by an Arduino (Bpod Generation 2 and associated code in Matlab 2019). Odorants (ethyl acetate, 2-pentanone) were diluted into approximately 4% v/v mineral oil and were delivered to mice via a Teflon odour tube placed in front of the nose of the mouse. Clean air was flowed through the odour tube continuously and odorants were delivered by programmatically mixing a given odorant into the airstream for a duration of up to 1.5 s. Mice were head-restrained to the behavioural apparatus and placed via a magnetic base such that the two reward delivery spouts lay equidistant below and in front of their mouths. Spout positions were finely adjusted to maintain equidistance for each mouse.

Once established on food and water restriction, mice began behavioural training across two phases. In the first phase, food- and water-restricted mice learned to voluntarily lick spouts to receive either a food or water reward, with both rewards equally available. A lick detected at the food spout resulted in a ~5-μl food reward, and a lick detected at the water spout resulted in a ~5-µl water reward. Each trial was followed by an inter-trial interval (ITI) of 1–3 s, with a maximum trial time of 10 s (in the event that no lick occurred). Mice performed this simplified two-reward collection task until they proficiently (within 1 h) collected sufficient food and water rewards to reach satiation. During this first training phase, the ITI was gradually increased from 1 s to 3 s. Following proficiency in the first training phase, mice were introduced to the full task structure as the second training phase. Following a variable ITI period (minimum 2 s, maximum 8 s, uniformly distributed), either a Go or No-Go odour was presented to the mouse for a maximum of 1.5 s; odours were terminated immediately following a detected lick to either spout. Mice that licked during the Go-odour period to either the food or water spout were rewarded with food or water from that spout (~5 µl). Licks made during the No-Go-odour period resulted in a longer ITI period and were not rewarded. Mice were trained until they consistently obtained sufficient food and water rewards to satiate both needs, reliably responded to the Go odour when hungry or thirsty, withheld licking during the baseline pre-odour period, and correctly rejected responses to the No-Go odour (>90% correct rejection rate). Data were collected from mice with behaviour sessions following the same task structure as that described for the second training phase.

We empirically chose the food (liquid Ensure with added salt) and water rewards to reduce cross-talk between needs by minimizing the extent to which a food reward would decrease thirst. The added salt additionally reduces the hedonic value of the food reward, as mice will not consume it when not hungry (Extended Data Fig. 1b) but will consume plain Ensure in the absence of hunger (data not shown). It is possible that the extra salt content of the liquid food reward leads to an increase in thirst over time. However, on the timescales of Buridan’s assay, there does not appear to be a link between the amount of food rewards collected and the subsequent collection of water rewards: there was no significant timing relationship between food choices and water choices (Extended Data Fig. 1e), nor any relationship between the amount of salted liquid food rewards collected in a bout and the amount of water collected in the subsequent bout (Extended Data Fig. 1f). This suggests that the switching behaviour we observe cannot be simply accounted for by fast-timescale induction of thirst from the salty food.

We note that at the start of the assay, our mice are not usually exactly equally thirsty and hungry. However, while performing the assay, the mice often encounter being approximately ‘equally thirsty and hungry’ according to our quantitative behavioural definition of thirst and hunger. That is, they will experience as many food-collecting trials as water-collecting trials until they reach satiation for both (see Extended Data Fig. 1d for an example). Note also that our Buridan’s assay is distinct from ‘Buridan’s paradigm’, a visuomotor task in *Drosophila* mimicking a state of indecision not involving a choice between needs^52^.

### Optogenetics behavioural experiments

*Rxfp1-P2A-cre* mice maintained with ad lib food and water access were screened after at least 2 weeks of viral expression for optogenetically induced drinking behaviour in their home cages (stimulus paradigm: 30-s on, 30-s off, 20-Hz stimulation with a 450-nm laser (Doric), pulse width 20 ms, measured at 15 mW at the end of the fibre optic cable). Mice with clear optogenetically induced drinking behaviour were used for subsequent experiments in Buridan’s assay; mice with no clear optogenetically induced drinking (probably owing to a lack of sufficient transduced cells or a misalignment of the optogenetic fibre with transduced cells) were discontinued from further study. Following behavioural training, mice were returned to ad lib food and water (sated condition) prior to optogenetic experiments. Mice performed Buridan’s assay while sated (Fig. 1m) or food-restricted (Fig. 1n) and received 20 stimulation epochs, each lasting 10 s at 20 Hz with 2-ms pulse widths of 450-nm, 15-mW laser light; epochs were repeated approximately every 2 min. Stimulation epochs were pseudorandomly triggered during the ITI phase of the assay.

### Freely moving behaviour for food versus water choice

In a freely moving version of Buridan’s assay, mice were food and water restricted, then placed in a four-sided custom operant chamber (Panlab, Harvard Apparatus) containing two levers and two corresponding reward ports delivering incremental salted liquid food (in the freely moving assay, liquid food was Soylent salted to 0.5 M NaCl concentration) or water. The levers and reward ports were arranged diagonally on opposite walls and mice were required to collect reward from a given port before more reward could be triggered at the same port (Extended Data Fig. 1i). Thus, to repeatedly collect rewards of a given type, mice had to run diagonally back and forth across the chamber, triggering reward (~5 µl) with a lever press and collecting it at the corresponding reward port. Unlike the head-fixed olfactory Go/No-Go task, the freely moving assay was conducted without any cue-based instrumental conditioning so that mice made free choices both for which reward to collect as well as when to collect a reward. Because mice passed through the centre of the arena after each reward collection, they were repeatedly equidistant from both food and water manipulanda. Behavioural session data (Extended Data Fig. 1j,k) were collected following several days of training in which mice became proficient at triggering and rapidly collecting reward for both reward types. Behavioural sessions typically lasted 1 h before satiation.

### Electrophysiological recordings

All recordings were acquired using Neuropixels 1.0 probes and associated hardware. Electrodes were cleaned prior to recordings with saturated Tergazyme detergent solution (Alconox), washed with pure water, and allowed to dry completely. Before each recording, electrode tips were coated in the fixable dye CM-DiI (Thermo Fisher) and dried. The Kwik-Cast coating over each craniotomy was removed and craniotomies were flushed with sterile saline prior to placing the mouse on the experimental apparatus. Once on the experimental apparatus, the reference and ground contacts of each probe were connected in circuit to each other and to the mouse’s reference electrode and headbar. In the case of optogenetic recording experiments, a fibre optic cable was connected to the fibre optic cannula on the mouse’s cranium. A circular positioning apparatus (Multi-Probe Manipulator, New Scale Technologies) was used to place four Neuropixels 1.0 probes above the mouse’s skull. Probes were positioned radially around the anterior–posterior axis (front left probe, –30°; front right probe, +30°; back left probe, –150°; back right probe, +150°). All probes were positioned at a + 15° angle from the dorsal–ventral axis. Micromanipulators (New Scale Technologies) were used to finely position probe tips at the surface of the brain for each craniotomy. The four probes were simultaneously inserted into the brain at a speed of 3.33 µm s^−1^. Insertion depths ranged from 3.85 mm to 6 mm but were generally between 4 and 5 mm from the brain surface. Following the completion of probe insertions, ~10 min were allowed to elapse before recording started to allow for any residual brain motion around the probes to settle. Data was acquired and written to disk using SpikeGLX (B. Karsh) using default settings (AP gain = 500, recordings acquired from the bottom 384 electrode sites per probe). Acquisitions across probes were synchronized using a square wave 0.5-s duration pulse with a 1-s period. The probe synchronization signal, behavioural signals, and any optogenetic stimulation signals were concurrently acquired on a Nidaq (Texas Instruments) and later aligned to the probe synchronization signal (TPrime, B. Karsh). Videos of the mouse’s face, head, and body were acquired during recordings and synchronized using infrared LEDs coupled to a trial-start TTL pulse recorded on the Nidaq.

In all experiments, the experimental setup period (prior to recording start) was conducted with spouts lowered away from the mouse’s mouth and odour airflow turned off. Just prior to the start of the behavioural assay, spouts were raised to an accessible position and odour airflow was turned on. Mice performed Buridan’s assay during recordings until satiation; subsequently, behavioural sessions were terminated and recording completed. In the case of optogenetic stimulation experiments performed during recording (Fig. 4k–o and Extended Data Fig. 10), spouts remained lowered and airflow remained off after the start of recording until the completion of optogenetic stimulation epochs (10-s stimulation at 20 Hz with 2-ms pulse widths of 15-mW 405-nm laser light, 20 stimulation epochs per session spaced 1 min apart); following a 5-min rest period, spouts were raised, airflow was turned on, and mice performed Buridan’s assay with no further optogenetic stimulation.

### Brain registration and electrode tracks reconstruction

Mice were euthanized following the completion of experiments and perfused with ice-cold phosphate buffered saline (1× PBS, Thermo Fisher) and 4% paraformaldehyde (PFA, Electron Microscopy Sciences). Brains were dissected from the skull and postfixed overnight in 4% PFA at 4 °C. Brains were cleared as previously described^16^. Following clearing, brains were imaged across both hemispheres in the horizontal plane on a LaVision light-sheet microscope in dibenzyl ether. Two image volumes were collected: a 488-nm autofluorescence volume and a 532-nm CM-DiI volume. Volumes were each collected at a 4-µm step size in the *z* axis and a 4-µm pixel size at 0.8× magnification using a single light-sheet horizontal focus.

Both resulting volumes were down-sampled to 25 µm. The 488-nm autofluorescent volume was registered using an affine transform followed by a warping b-spline transform (Elastix) to the Allen Brain Atlas CCFv3^53^ (available at https://allensdk.readthedocs.io/en/latest/). The resulting transformation was used to deform the 532-nm CM-DiI volume onto the reference atlas. Alignments between the reference atlas and both the autofluorescent volume and the CM-DiI volume were visually inspected for good agreement between structures. The Python image volume viewer Napari^54^ was used to label points along electrode tracts; each set of points per track was given a unique name and saved per brain. Custom Python code was used to transform probe point sets to insertion tracks and to map electrode locations to brain regions (see code repository on GitHub, https://github.com/erichamc/brainwide-npix). Using custom code, local field potential (LFP) data from each probe was extracted and plotted against colour-coded (following the Allen Institute Brain Atlas colour map) regional annotations, and fine adjustments were made to the position of the lowest point labelling a given trajectory until a satisfactory qualitative alignment between LFP activity and regional boundaries was observed (Extended Data Fig. 2d).

### Spike sorting and preprocessing

All recordings were pre-processed using the CatGT tool (B. Karsh, https://billkarsh.github.io/SpikeGLX/#catgt) to common average reference (CAR) recorded voltage traces per-probe and to zero out any transient electrical artifacts remaining after CAR (command-line option: -gfix=0.40,0.10,0.02). Following preprocessing with CatGT, data was spike sorted using Kilosort3 (https://github.com/MouseLand/Kilosort). Cluster spike times (output from Kilosort3) and Nidaq events (detected via CatGT) were aligned to the reference probe sync signal using TPrime (Karsh, https://billkarsh.github.io/SpikeGLX/#tprime). Cluster waveform averages were calculated using C_Waves (B. Karsh). Code from the ecephys_spike_sorting pipeline (J. Colonell, https://github.com/jenniferColonell/ecephys_spike_sorting) was used to organize pipeline executables and input/output files, and was further used to calculate QC metrics on Kilosort3 clusters and to tag candidate electrical noise clusters. Following all preprocessing, spike sorting, and postprocessing steps, all clusters were manually examined using Phy2 (https://github.com/cortex-lab/phy)^55^ and re-labelled as noise or non-noise clusters as necessary. An automated threshold was set for well-isolated units based on manual noise cluster labelling and QC metrics (inter-spike interval violations <0.1, signal-to-noise ratio >2, number of spikes per cluster >500). The combination of these thresholds qualitatively agreed well with manual annotation of well-isolated versus multi-unit activity clusters. All clusters that did not pass these thresholds were excluded from analysis.

### Analysis software

All data analysis was carried out using Python code in Jupyter IPython^56^ Notebooks. These analyses relied heavily on Numpy^57^, Scipy^58^, Pandas^59^, and Scikit-learn^60^. Computational simulations were composed using Jax^61^. Seaborn^62^ was used for bar plots, box-and-whisker plots and KDE plots. Matplotlib^63^ was used for all other plots. Statsmodels^64^ and Scipy were used for all statistical analyses that were not carried out using bootstrapping.

### Behavioural data analysis

#### Selectivity index

We calculated the selectivity index as a per-session average of reward choices (no. of cumulative water choices − no. of cumulative food choices)/(no. of cumulative water choices + no. of cumulative food choices) (Extended Data Fig. 1a).

#### Markov process choice persistence counts

The persistence count distribution of a two-state Markov process follows a geometric distribution. We fit geometric distributions to the persistence counts data using Scipy (Fig. 1g and Extended Data Fig. 1k).

#### Definition of behavioural thirst, hunger and relative need

We defined a per-trial measurement of behavioural thirst or hunger, respectively, as the total number of water or food rewards the mouse would collect in the entire session minus the current number of collected water or food rewards. We further normalized these ‘behavioural thirst’ or ‘behavioural hunger’ values by the median number of total water or food rewards, respectively, that mice on food and water restriction collect in Buridan’s assay. For example, behavioural thirst = (no. of total water rewards in the session − no. of water rewards collected up to the current trial)/(median of no. of total water rewards collected by an initially hungry and thirsty mouse in a session, calculated across all sessions). We then further defined the relative level of behavioural thirst and hunger as an index ranging from −1 (maximally hungry versus minimally thirsty) to +1 (minimally hungry versus maximally thirsty), which we refer to as the relative need of the mouse and calculated as [(behavioural thirst − behavioural hunger)/(behavioural thirst + behavioural hunger)] (Extended Data Fig. 1d).

#### Marginal and conditional per-trial probabilities

To analyse the marginal or conditional probabilities of per-trial choices, we first collated all trials from behavioural sessions into a per-trial outcome table, in which each Go-trial was tagged with the position in session, previous Go-trial choice, subsequent Go-trial choice, cumulative rewards per session, and current number of food and water rewards collected. The marginal probability of choosing water on any given trial was fit using linear regression to predict rewarded Go-trial outcomes from the relative need value, for trials of sessions in which the mouse was under both food and water restriction (Fig. 1h). 95% confidence intervals on these fits were estimated by bootstrapping. We also calculated maximum likelihood estimates (MLE) for the marginal probability of choosing water on a rewarded trial as the fraction of rewarded trials in which the mouse chose water, evaluated for trials falling within a given 5-percentile-wide bin of relative need values (Fig. 1h, black dots). 95% confidence intervals on the MLE estimates were bootstrapped and plotted as vertical lines.

Using the tabulated trial choice outcomes and their associated previous trial or subsequent trial choice outcomes, we calculated an MLE Markov transition matrix for trials from all behavioural sessions. For the transition matrix given relatively balanced needs (Fig. 1k), we used only trials with a relative need value between –0.25 and +0.25. We excluded sessions from these analyses in which mice were only under a single restriction paradigm (food only or water only), and we excluded trials in which the mouse had fewer than 10 remaining rewards to collect of a given type (to avoid sampling issues). The self-transition probability for food choices and water choices (Fig. 1j) was also estimated by fitting a linear regression on relative need values per trial to predict whether a food choice would follow a previous food choice; an equivalent procedure was applied for water trials. The 95% confidence intervals were estimated for each self-transition probability fit by bootstrapping. MLE values for the probability of self-transition were calculated as the fraction of self-transitions for a given choice type, restricted to trials whose relative need value fell within a given 5-percentile-wide bucket, with 95% confidence intervals for MLE values estimated by bootstrapping.

#### Comparison between behavioural features predicting upcoming choice

We compared the upcoming-choice predictiveness of needs and of previous choice by fitting and evaluating a support vector machine with a radial basis kernel (L2 regularization weight C = 1.0 and gamma scaled according to the feature variance; Scikit-Learn defaults with gamma = ‘scale’). When evaluating the predictiveness of needs, we fit a two-feature model using only the behavioural thirst and behavioural hunger (see above) values of trials in a 50% training split of the data. When evaluating the predictiveness of previous choice, we fit the support vector machine using only the binary outcome of the previous choice to predict the present choice in a 50% training split of the data. Predictiveness (AUC) was evaluated on test data for models fit separately on each session, yielding median and 95th percentile confidence interval values for each parameter set across all sessions (*n* = 15 mice, 22 sessions).

#### Optogenetic behavioural experiments

For all optogenetic stimulation epochs, nearby Go-trial start times were tagged by the choice outcome (food, water, miss) and the start time relative to the nearest optogenetic stimulation epoch onset time. These food and water choice trial times, relative to stimulation epoch onset, were smoothed across time using a kernel density estimator (KDE) (Seaborn, Scipy) to yield a probability density estimate of a food or water choice response as a function of time relative to optogenetic stimulation onset. For sated mice, no food choices were made, therefore the KDE analysis was omitted.

### Electrophysiological data analysis

#### Firing rates

Spikes for each neuron were binned at 10-ms resolution and the binned counts were divided by the bin width and causally smoothed using a forward moving average window of 100-ms to yield smoothed firing rates at a 10-ms resolution. These rates were *z*-scored across the duration of a session within neuron. For trial-timing relative analyses, the *z*-scored rates were concatenated into a per-trial vector 410 bins long (4.1 s, the minimum trial time) and aligned to a trial-start trigger signal (recorded on the Nidaq and corrected into the reference probe synchronization time) such that the first bin per trial corresponded to the 10 ms adjacent to the trial-start trigger time. The baseline activity rate per trial was defined as the average number of spikes for a given neuron in the 1 s before odour onset.

#### Regional analyses

All analyses of single neurons used well-isolated clusters identified by Kilosort3 and postprocessing analyses (see ‘Spike sorting and preprocessing’). Each neuron was tagged with a corresponding anatomical location using the atlas-aligned location of the electrode at which the neuron’s detected waveform had the greatest amplitude. These anatomical locations were used to extract from the Allen Institute CCFv3 annotation volume and associated structure tree a corresponding region name. Depending on the level of analysis, the region name used was either the leaf node of the structure tree or a higher order structure. In all figures, regions are colour-coded following the colormap convention set by the Allen Institute’s Mouse Brain Atlas and were extracted using the AllenSDK.

#### Significantly modulated cells

The following analyses of single neurons used only significantly task- and state-modulated cells: Fig. 2e,f and Extended Data Figs. 3–5. Significant modulation was defined as a logical OR operation over five measures each assessed by two-sided *t*-tests: difference in average firing rate in the 1 s before odour onset (baseline firing rate) between food choice trials and water choice trials; difference in baseline firing rate between Go trial responses (hit trials) and Go trial non-responses (miss trials); and differences between baseline firing rate and the average firing rate 0–1, 1–2, and 2–3 s after odour onset. *P* values for each measure of modulation were Benjamini–Hochberg false-discovery rate corrected for the total number of cells. This measure of significance was used as a pre-filter on cells; subsequent analyses used additional measures of significance against relevant null distributions (see below on null distributions).

#### Visualization of baseline firing rates

Neurons were sorted by the correlation of the upcoming choice identity and their average baseline firing rate in the 1 s prior to odour onset and visualized with average firing rates in the 1 s before each trial concatenated together (Fig. 2c).

#### Agglomerative clustering

For significantly modulated cells, per-trial firing rates were averaged within condition (food choice, water choice, miss/sated) and condition-averaged firing rate vectors were concatenated. These concatenated, per-cell condition-averaged rates were treated as multidimensional measurements where each concatenated firing rate bin was a feature. Using the library Scanpy^65^, the cell by feature matrix was first reduced in dimension using principal components analysis, then a neighbourhood graph of observations was computed using *n* = 5 neighbours, then a uniform manifold approximation and projection^66^ manifold was computed, and finally clusters were identified on this manifold using Leiden clustering^67^. Cells were ordered by these cluster identities and their condition-averaged *z*-scored firing rates were visualized (Extended Data Fig. 3a,b).

#### Variance explained by regressors

A series of binary regressors were constructed from behavioural variables for each trial: choice outcome (food versus water); early (first third of trials in a session) versus late (last third) of food choices; early (first third) versus late (last third) of water choices; hit versus miss; Go-odour trial versus No-Go-odour trial. From these regressors, a set of 8 measurements of firing rate variance explained were made from each cell’s per-trial activity: average baseline activity (1 s pre-odour) compared to choice outcome, early versus late food trials, early versus late water trials, and hit versus miss regressors; average odour activity (300-ms window following odour onset) compared to Go versus No-Go and choice outcome regressors; and average response activity (1-s activity window starting 1 s post-odour) compared to choice outcome and Go versus No-Go regressors (Extended Data Fig. 3c). For regional analyses of variance explained, distributions of variance explained by neuron within a region were visualized for a given regressor. Regions were sorted by the average value of firing rate variance explained per neuron recorded in that region, for regions with greater than 30 recorded neurons.

#### Null distributions for single-cell analyses

For all single-cell regression analyses, a per-cell null distribution was constructed, and true measurements for each cell (for example, variance explained for a given regressor) was compared to the corresponding null, with significance determined by the resulting one-sided tail statistic with *P* ≤ 0.05 as the threshold. For the variance explained measurements, each cell’s null distribution was constructed by circularly permuting the firing rate time series with respect to the regressor time series. We note that significance tests against null distributions with random (unstructured) permutation of the time series failed to remove spurious long-timescale correlations, though we also note that long-timescale correlations may be relevant to some of the state phenomena we are interested in and therefore circular permutation may be overly conservative; future studies that enable tracking the same neurons across multiple sessions should increase the statistical power of the corresponding null distributions. For analyses that considered the average variance explained per region, an additional null distribution bootstrapping mean values from the circularly permuted null for cells in each region was used (dashed lines in Fig. 2e and Extended Data Fig. 5).

The following *P* values for regional means of variance explained are associated with Fig. 2e and Extended Data Fig. 5c and were obtained by bootstrapping (10,000 samples) the mean per-cell variance explained value within region and comparing the one-sided tail statistic to a similarly bootstrapped regional null using the per-cell null distribution variance explained values (obtained as described above). These *P* values were then FDR-corrected for multiple comparisons across brain regions: MRN, 0.000; SCiw, 0.000; APN, 0.000; CP, 0.000; OLF, 0.000; FF, 0.000; FS, 0.000; ORBl5, 0.000; VTA, 0.006; ACB, 0.008; PeF, 0.010; LHA, 0.016; ORBl1, 0.124; AIv5, 0.127; ORBl2/3, 0.130; SSp-m6a, 0.130; ORBl6a, 0.130; ZI, 0.130; AON, 0.272; PO, 0.279; VPM, 0.279; VAL, 0.279; RN, 0.279; SI, 0.279; AAA, 0.327; EPd, 0.336; VM, 0.417; AId6a, 0.520; SCig, 0.603; CA1, 0.748; MOs6a, 0.828; POST, 0.834; LP, 0.834; PRNr, 0.955; PPN, 1.000 (brain region abbreviations are provided in Extended Data Table 1).

#### Null distributions for population predictions

For analysis of population predictiveness of the upcoming choice, which is potentially confounded by long-timescale correlations in the neural activity, we took two approaches: (1) we compared all analyses to both a circularly permuted null distribution and to a session-permuted null distribution (the two dashed lines in Fig. 2g; both gave similar results); and (2) we evaluated predictiveness on a set of held-out test trial data flanking behavioural switches (reward trials –5 to –2 and reward trials +2 to +5 relative to the first trial with an altered reward choice as trial 0); because this test set paired choices of each reward type to an equivalent set nearby in time, it helped to eliminate the confound of spurious long-timescale correlations with the choice.

#### Variance explained co-occurrence

An 8 × 8 regressor information correlation coefficient matrix was calculated from the Regressor variance explained × Cell matrix. Euclidean pairwise distances were evaluated between the resulting correlation coefficient values and a linkage between distances was subsequently estimated. Entries of the correlation coefficient matrix were reordered according to the leaves of the hierarchical linkage (Extended Data Fig. 3e).

#### Coupling analyses

Cells were pooled across all recordings (*n* = 7 mice or recordings, number of cells per region given in Extended Data Table 1). For each cell, firing rates binned at 10 ms in the 1 s pre-odour baseline period of each Go-trial were baseline-subtracted by the cross-session mean firing rate in the given choice outcome and then concatenated together for trials across the session. A matrix of pairwise noise correlations between cells was then calculated using these concatenated firing rates. Cells were categorized as goal-significant or non-goal-significant as described above (null distributions for single-cell analyses), and the noise correlation matrix was sliced according to the categories given in Extended Data Fig. 3q,r to yield the distributions plotted.

#### Population decoding

All population choice prediction analyses were performed using a linear discriminant analysis (LDA) classifier with shrinkage of the covariance matrix determined analytically. All quantifications of model predictive accuracy (receiver operating characteristic (ROC) AUC) were evaluated on held-out test data. Feature vectors for population decoding used either single-time bin activity vectors for simultaneously recorded neurons (Fig. 4e and Extended Data Fig. 8b) or activity vectors averaging activity per-neuron in the 1-s pre-odour baseline period (Figs. 2g and 4c and Extended Data Fig. 8a). For upcoming choice classifiers (‘goal dimension’ classifiers), trials in which licking occurred during the pre-odour baseline period were excluded to avoid behavioural contamination. Visualizations of neural activity along the discriminating axis used the decision function of a logistic regression classifier with inverse regularization strength set to 0.02. In the case of optogenetic experiments, a goal dimension was constructed using the difference of average water and food trial baseline activity during the behavioural assay, with weights normalized by the summed standard deviation within trials of each outcome^39^. One-second-binned neural activity surrounding each optogenetic stimulation epoch (–10 s to +20 s) was projected onto this goal dimension (Fig. 4i–o and Extended Data Fig. 10f,g). Alternatively (Extended Data Fig. 10c–e), a ‘thirst-stim’ classifier was constructed using average activity during optogenetic stimulation (averaged across 10 s of stimulation) or average activity in the 3 s prior to stimulation onset to predict stimulation versus non-stimulation periods, respectively. Projection of neural activity during behaviour onto this ‘thirst-stim’ dimension was used to measure activity along this optogenetic thirst dimension during different behavioural epochs.

#### Prediction of upcoming switches

An upcoming choice classifier using simultaneously recorded population activity was constructed using logistic regression with linear features consisting of the average neural activity per cell in the 1 s before odour onset. Regularization strength was set to 0.05. The classifier was trained on 70% of rewarded trials, with 30% held out. The decision function of this classifier was then evaluated on all trials across each session to yield a population activity projection along the goal dimension. To predict the probability of switching on a given trial, the distance from zero of the magnitude of the upcoming choice decision function, evaluated on 1 s pre-odour activity, was linearly rescaled into a probability using a unidimensional LDA classifier fit on the switch outcomes of all trials (Fig. 4g,h). To measure the performance of the goal activity projection in predicting upcoming switches across a session, the receiver operating characteristic AUC was measured directly using the distance from zero of the magnitude of the upcoming choice decision function as evaluated on 1-s pre-odour activity across all rewarded trials (Fig. 4i,j).

#### Post-odour choice-selection activity analyses

To assess information about choice gained following odour onset, population decoders of choice were constructed on a per time bin basis using baseline-subtracted firing rate activity in that time bin (Extended Data Fig. 11a). The baseline activity removed from each time bin consisted of the average activity in the 1 s before odour onset, evaluated on a per-trial basis. Removing the baseline activity per cell removes the average baseline predictiveness of upcoming choice, such that predictiveness increases can only come from changes in neural activity beyond the average baseline rate per trial. Classifiers for each region were constructed using the simultaneously recorded population activity vector within a given region (Extended Data Fig. 11a). The visualized baseline-subtracted post-odour predictiveness used the AUC as evaluated first within session on held-out test trials, then averaged across session replicates. Regions without multiple session replicates or less than 30 neurons are marked with an asterisk.

To assess whether fluctuations of the baseline goal activity modulated post-odour choice-selection dynamics, we constructed classifiers of choice using simultaneously recorded firing rate activity during the 1–2 s post-odour period from all simultaneously recorded neurons (Extended Data Fig. 11b,c) or from neurons in a given region (Extended Data Fig. 11f). For these analyses, we used a logistic regression classifier with linear features and a regularization factor of 0.1 (Scikit Learn). Classifiers were trained on a training dataset of 50% of trials and subsequent projections were evaluated on the 50% test set of trials. Projections to the discriminating choice-selection dimension were constructed using the per-region classifier decision function, which we further baseline-subtracted using the per-trial projection values in the 100 ms prior to odour onset. For each corresponding choice trial in the behavioural session, we computed baseline activity in the goal dimension (using the decision function of a logistic regression classifier trained to predict upcoming choice from the 1 s pre-odour neural activity of all simultaneously recorded neurons across regions). Both activity in the goal dimension and activity in the choice-selection dimension were normalized from –1 to 1 using a secondary linear classifier function to remove systematic differences in decision function magnitudes between behavioural sessions. For the analyses presented in Extended Data Fig. 11b,d,f, projections onto the choice-selection dimension were binned into 0–33, 34–66, and 67–100 percentile groups using the per-trial baseline goal dimension activity, with percentiles calculated within session and within choice. Linear regression analyses (Extended Data Fig. 11c,e) used per-trial goal dimension activity magnitude directly, not percentiles. For the regression analysis of the modulation of post-odour choice-selection activity dynamics by within-choice baseline goal activity, choice-selection activity was summed from 0.1 s post-odour to 0.9 s post-odour.

### Video decoding analysis

Videos of the face and body, acquired at 100 frames per second each, were cropped to regions of interest (ROIs) and converted to greyscale from RGB. ROIs were converted to motion energy (change in pixel intensity from frame to frame) and the top 500 principal components were extracted for each ROI by singular value decomposition using FaceMap^68^. Principal component video data was synchronized to behaviour data using a small in-frame LED trigger signal at the beginning of each behavioural trial. Behavioural decoding and decoding performance evaluation was performed using LDA with receiver operator characteristic area under the curve (ROC AUC) quantification using an equivalent approach to that used for decoding of neural data.

### Mathematical modelling

The mathematical model consists of several coupled differential and stochastic differential equations. These equations describe the shape of an energy landscape of needs as a function of thirst and hunger magnitudes; the Langevin dynamics of motion across the landscape as a function of time; and the update dynamics for thirst and hunger magnitudes as a function of odour presentation (sampling times) and the current position on the landscape (behavioural choice events) (Fig. 3b and Extended Data Fig. 6c–f). We chose a simple three-dimensional energy landscape shape (a two-dimensional space with a landscape depth defined at every position) that placed harmonic wells centred at three locations at equilateral distance to each other; these locations represent the means of thirst-related, hunger-related, and other needs-related neural activity. To give smooth saddles between wells we expressed the landscape as the log-sum of Gaussian probability density functions (Extended Data Fig. 6e,f) according to the following equation:

1 $$U(x,t)=\log (s\times T(t)\times {\Phi }_{{\rm{w}}}(x)+s\times H(t){\times \Phi }_{{\rm{f}}}(x){+\Phi }_{{\rm{o}}}(x))$$

where the shape of each well is defined by the following negative probability density functions of multivariate normal distributions:

$${\Phi }_{{\rm{w}}}(x)=\Phi (x\,;{\mu }_{{\rm{w}}},\Sigma )$$

$${\Phi }_{{\rm{f}}}(x)=\Phi (x\,;{\mu }_{{\rm{f}}},\Sigma )$$

$${\Phi }_{{\rm{o}}}(x)=\Phi (x\,;{\mu }_{{\rm{o}}},\Sigma )$$

and where *μ*_w_ is the centre of the well for the thirst-related space; *μ*_f_ is the centre of the well for the hunger-related space; *μ*_o_ is the centre of the well for the ‘other needs’-related space; $\Sigma ={\sigma }^{2}\times I$ is the covariance matrix of each normal distribution and is equivalent between all wells; $T(t)$ is the magnitude of thirst at time *t*; *H*(*t*) is the magnitude of hunger at time *t*; and *s* is a scaling factor that aligns experimentally observed normalized thirst and hunger magnitudes to the appropriate scale within the model. Thus, as the thirst and hunger magnitudes change over time, the depth of the respective wells in the energy landscape are scaled, leading to varying gradients on the landscape over time as a function of each need (Fig. 3b,c, Extended Data Fig. 6c–f and Supplementary Video 1). For simplicity, we assumed a constant scale of other needs, such that thirst and hunger are given as relative magnitudes to other needs.

Behaviour of neural activity on this simplified two-dimensional subspace is approximated by overdamped Langevin dynamics in the following equation (see also Extended Data Fig. 6c,d):

2 $$x(t+1)=x(t)+{\rm{d}}t\times g\times -{\rm{\nabla }}U(x,t)+\sqrt{{\rm{d}}t}\times n\times N(0\,;I)$$

At every discretized time step, we add to the current position *x*(*t*) the negative gradient of the present energy landscape, scaled by the factor *g*, and a time-independent white noise *N* (zero-centred normal distribution with covariance *I* the identity matrix, indicating zero dependence between noise in each dimension) scaled by a noise factor *n*. Behaviour of motion on the landscape is heavily dependent on the relationship between *g* and *n* and their magnitudes with respect to the distances between well centres and the scale on the well shapes. Higher values of *n* drive more frequent transitions between spaces on the landscape and in the limit overwhelm the contribution of the energy landscape to the dynamics; higher values of *g* increase the dominance of the energy landscape over the dynamics contributed by noise and decrease the transition frequency, as movement on the landscape tends to be pulled towards the closest energy well. The scale factor *s* in *U*(*x*, *t*) modifies the propensity of the system to stay in the food or water seeking zones instead of the ‘other needs’ zone. Increasing magnitudes of both *s* and *g *increase the rapidity of transitions between zones.

Behavioural emissions from these dynamics are given by a partitioning of the subspace into zones according to which unscaled negative probability density function $\phi$ has greater magnitude at every position, yielding a standard maximum likelihood decision function. We choose this maximum likelihood partition for simplicity, but we propose that learning in a particular environment or context can shape more arbitrarily complicated partitions of this subspace with respect to behaviour. Thus, behavioural choice emissions are defined by position *x*(*t*) and a sampling-cue at time *t*′ with the following equation:

3 $$b(x,{t}^{{\prime} })=\left\{\begin{array}{l}{\rm{miss}},{\Phi }_{{\rm{o}}}(x\,;{t}^{{\prime} })\le {\Phi }_{{\rm{w}}}(x\,;{t}^{{\prime} })\,{\rm{and}}\,{\Phi }_{{\rm{o}}}(x\,;{t}^{{\prime} })\le {\Phi }_{{\rm{f}}}(x\,;{t}^{{\prime} })\\ {\rm{food}},{\rm{not}}\,{\rm{miss}}\,{\rm{and}}\,{\Phi }_{{\rm{f}}}(x\,;{t}^{{\prime} }) < {\Phi }_{{\rm{w}}}(x\,;{t}^{{\prime} })\\ {\rm{water}},{\rm{not}}\,{\rm{miss}}\,{\rm{and}}\,{\Phi }_{{\rm{w}}}(x\,;{t}^{{\prime} })\le {\Phi }_{{\rm{f}}}(x\,;{t}^{{\prime} })\,\end{array}\right.$$

The magnitude of the thirst *T*(*t*) and hunger *H*(*t*) are decremented by a fixed amount *r*_w_ and *r*_f_, respectively, and fixed feedback delay *l* after food or water choices are made, according to the following equations:

4 $$\frac{{\rm{d}}T}{{\rm{d}}t}=\left\{\begin{array}{c}0,b(x,t-l)\ne {\rm{water}}\\ {r}_{{\rm{w}}},b(x,t-l)\equiv {\rm{water}}\end{array}\right.$$

5 $$\frac{{\rm{d}}H}{{\rm{d}}t}=\left\{\begin{array}{c}0,b(x,t-l)\ne {\rm{food}}\\ {r}_{{\rm{f}}},b(x,t-l)\equiv {\rm{food}}\end{array}\right.$$

*r*_w_ and *r*_f_ correspond to the reward size given to the mouse and approximate the incremental changes in needs upon reward collection; we set these values based on high initial thirst *T*_0_ and hunger *H*_0_ values such that simulated behaviour in Buridan’s assay resulted in similar numbers of cumulative rewards collected until satiation (predominantly ‘miss’ outcomes) as experimental behaviour. We set the feedback delay *l* to 2 min in simulation time based on a rough review of the literature^7^ but did not fine-tune the value based on experimental data. For simplicity, we did not include any hysteresis in the update to the thirst and hunger magnitudes, excepting a fixed delay time in the update. We note that a more detailed model may include hysteresis, accounting, for instance, for the rate at which rewards are collected or characteristic dynamics to the rate at which the landscape can change; possible anticipatory dynamics in the sensation of homeostatic deficits; and more complex interactions between the rewards and needs (for example, if a reward decreases one need but increases the other on longer timescales, as may be the case for the salted ensure). All visualizations or measurements of simulated activity along the goal dimension use the projection of the current position onto the unit vector given by

$$\frac{{\mu }_{{\rm{w}}}-{\mu }_{{\rm{f}}}}{\Vert {\mu }_{{\rm{w}}}-{\mu }_{{\rm{f}}}\Vert }$$

which in the case of our parameters is simply $$\left[\begin{array}{c}0\\ 1\end{array}\right]$$.

### Model fitting and derivations

The mathematical model described above has several interrelated fixed parameters whose values alter the system behaviour across a simulated behavioural session. We sought a set of parameters whose values best produce simulations with summary statistics matching those of the behavioural experiments. We chose to fit the model to the behavioural data, rather than the neural data, for two reasons: (1) so that we could evaluate the extent to which the model matches and predicts behavioural results, independent of the constraints of the neural data (Fig. 3); and (2) so that we could evaluate the extent to which this class of model, tuned to the behavioural outcomes, would anticipate aspects of the neural data (Fig. 4). Since the model we present is an equation of motion that enables a discretized, stochastic forward simulation of behavioural sessions from an initial position and initial thirst and hunger magnitudes, and any given session simulation for a set of parameters will differ across repeated simulation runs, the output of the model cannot be directly tuned to match the trajectories of behavioural sessions. Fitting is additionally complicated from a computational perspective by the fact that the model is discretized to a fine temporal time step (1/100th of a second), so that ~1,000 model steps are evaluated between every simulated trial.

An optimal model fitting procedure maximizes the likelihood of the observed data given predictions from the model and its parameters, subject to any constraints. To perform this optimization, we exploited several aspects of the experimental data and model:

First, recognizing that the behavioural sessions exhibited a strong Markov property (conditional independence), we transformed behavioural sessions (chains of 100 s of trials) into a collection of pairs of sequential rewarded trial outcomes, for example, food-to-food, food-to-water, water-to-food, and water-to-water, and we tagged each trial by their current thirst and hunger magnitudes and the time elapsed between reward choices. We then considered each pair of trials independent from all other pairs of trials. The problem of optimizing the model parameters then becomes a problem of jointly optimizing the probability of each trial pair given the model and parameter set.

Second, while the stochastic differential equation described above does not give a probability estimate for this trial-pair data, results from the field of non-equilibrium statistical mechanics and transition state theory give approximate equations related to the transition probability rate between states for Langevin equations with a form similar to what we describe above. Specifically, we adapted Kramers’ first passage problem^33,34^, which describes the escape rate over an energy barrier for a diffusive particle in a harmonic well, to give a theoretical expression for the transition probabilities between choices as a function of thirst, hunger, and time between trials. This theoretical expression utilizes the same model parameters and energy landscape as the forward equation of motion and thus links the model parameters to the observed behavioural events. We additionally exploited equilibrium relationships between the landscape and noise to constrain the fitting. We provide the derivation of these theoretical expressions as follows.

#### Derivation of the state transition probability equation

Following transition state theory, we can write down a set of general time-dependent differential equations for the probability that the system will be in one state or another (in our case, the zone around the food well or the zone around the water well):

6 $$\frac{{\rm{d}}}{{\rm{d}}t}{P}_{{\rm{w}}}(t)={P}_{{\rm{f}}}\times {\omega }_{{\rm{fw}}}-{P}_{{\rm{w}}}\times {\omega }_{{\rm{wf}}}$$

7 $$\frac{{\rm{d}}}{{\rm{d}}t}{P}_{{\rm{f}}}(t)={P}_{{\rm{w}}}\times {\omega }_{{\rm{wf}}}-{P}_{{\rm{f}}}\times {\omega }_{{\rm{fw}}}$$

where *P*_w_ is the probability of being in the water zone; *P*_f_ is the probability of being in the food zone; *ω*_fw_ is a transition probability rate from the food zone to the water zone; and *ω*_wf_ is a transition probability rate from the water zone to the food zone. As a simplifying assumption we ignore transitions to the ‘other needs’ zone and will consider only trials with no misses between rewards. Therefore,

8 $$\frac{{\rm{d}}}{{\rm{d}}t}{P}_{{\rm{w}}}({\rm{t}})=-\frac{{\rm{d}}}{{\rm{d}}t}{P}_{{\rm{f}}}(t),$$

and

9 $${P}_{{\rm{w}}}(t)+{P}_{{\rm{f}}}(t)=1$$

Conditioning on an observation of a previous food or water choice as time *t* = 0 gives the boundary conditions:

10 $${P}_{{\rm{w}}}(0)=1\,{\rm{and}}\,{P}_{{\rm{f}}}(0)=0\,({\rm{previous}}\,{\rm{choice}}\,{\rm{of}}\,{\rm{water}})$$

11 $${P}_{{\rm{w}}}(0)=0\,{\rm{a}}{\rm{n}}{\rm{d}}\,{P}_{{\rm{f}}}(0)=1\,({\rm{p}}{\rm{r}}{\rm{e}}{\rm{v}}{\rm{i}}{\rm{o}}{\rm{u}}{\rm{s}}\,{\rm{c}}{\rm{h}}{\rm{o}}{\rm{i}}{\rm{c}}{\rm{e}}\,{\rm{o}}{\rm{f}}\,{\rm{f}}{\rm{o}}{\rm{o}}{\rm{d}})$$

and therefore *P*_w_(*t*) becomes a self-transition probability *P*_ww_(*t*), the probability of being in the water zone at time *t* following being in the water zone at time *t* = 0, and equivalently for *P*_f_ (*t*) as self-transition probability *P*_ff_(*t*) for the probability of being in the food zone at time *t* following being in the food zone at time *t* = 0. Using equation (9) and substituting yields:

12 $$\frac{{\rm{d}}}{{\rm{d}}t}{P}_{{\rm{ww}}}(t)=(1-{P}_{{\rm{ww}}}\left(t\right))\times {\omega }_{{\rm{fw}}}-{P}_{{\rm{ww}}}\times {\omega }_{{\rm{wf}}}$$

13 $$\frac{{\rm{d}}}{{\rm{d}}t}{P}_{{\rm{ff}}}(t)=(1-{P}_{{\rm{ff}}}\left(t\right))\times {\omega }_{{\rm{wf}}}-{P}_{{\rm{ff}}}\times {\omega }_{{\rm{fw}}}$$

and the transition probabilities for switching are:

14 $${P}_{{\rm{wf}}}(t)=1-{P}_{{\rm{ww}}}(t)\,{\rm{for}}\,{\rm{a}}\,{\rm{water}}\,{\rm{to}}\,{\rm{food}}\,{\rm{transition}},$$

and

15 $${P}_{{\rm{fw}}}(t)=1-{P}_{{\rm{ff}}}(t)\,{\rm{for}}\,{\rm{a}}\,{\rm{food}}\,{\rm{to}}\,{\rm{water}}\,{\rm{transition}}.$$

Integrating equations (12) and (13) and solving the initial value problem gives:

16 $${P}_{{\rm{ww}}}\left(t\right)=\left(1-\frac{{\omega }_{{\rm{fw}}}}{{\omega }_{{\rm{wf}}}+{\omega }_{{\rm{fw}}}}\right)\times {{\rm{e}}}^{-\left({\omega }_{{\rm{wf}}}+{\omega }_{{\rm{fw}}}\right)\times t}+\frac{{\omega }_{{\rm{fw}}}}{{\omega }_{{\rm{wf}}}+{\omega }_{{\rm{fw}}}},$$

and

17 $${P}_{{\rm{ff}}}\left(t\right)=\left(1-\frac{{\omega }_{{\rm{wf}}}}{{\omega }_{{\rm{wf}}}+{\omega }_{{\rm{fw}}}}\right)\times {{\rm{e}}}^{-\left({\omega }_{{\rm{wf}}}+{\omega }_{{\rm{fw}}}\right)\times t}+\frac{{\omega }_{{\rm{wf}}}}{{\omega }_{{\rm{wf}}}+{\omega }_{{\rm{fw}}}}.$$

This yields the full time-dependent transition matrix:

18 $${P}_{{\rm{trans}}}(t)=\left[\begin{array}{cc}{P}_{{\rm{ff}}}\left(t\right) & {P}_{{\rm{fw}}}\left(t\right)\\ {P}_{{\rm{wf}}}\left(t\right) & {P}_{{\rm{ww}}}\left(t\right)\end{array}\right]$$

To evaluate these probability expressions, we need to specify the rate equations for *ω*_fw_ and *ω*_wf_. Kramers’ first passage problem describes the average time it takes for a particle residing within a harmonic potential well to first escape over an energy barrier; if the other side of the barrier is a second potential well with high barriers^34^, then the inverse of this first escape time is approximately the transition rate *ω*. For a one-dimensional Smoluchowski equation satisfying the fluctuation dissipation theorem^34^, this transition rate from state A to B has the form:

19 $${\omega }_{{\rm{A}}{\rm{B}}}\sim \left(\frac{\sqrt{{\nu }_{{\rm{A}}}\times {\nu }_{\ddagger }}}{2\times \pi \times \gamma }\right){{\rm{e}}}^{-\frac{{U}^{\ddagger }-{U}^{{\rm{A}}}}{{K}_{{\rm{b}}}{\rm{T}}}},$$

where ${U}^{\ddagger }$ is the potential evaluated at the transition state (saddle between wells); *U*^A^ is the potential evaluated at the minimum (well centre) of the source state; *K*_b_T is the Boltzmann constant multiplied by temperature, *γ* is the friction term, *ν*_A_ is the approximated frequency of the harmonic well at its centre, and *ν*_*t*_ is the approximated frequency of the transition state. To utilize this equation, we construe the equation of motion of our model as a one-dimensional Smoluchowski equation along the *y* axis (line between food and water wells). We set the friction term *γ* = 1, the noise term $n\approx \sqrt{{K}_{{\rm{b}}}{\rm{T}}}$, and we consider the gradient scale *g* as a scale factor on the energy landscape, rather than a scale factor on the gradient of the landscape in the equation of motion (yielding an equivalent effect on the gradient as it would have in the equation of motion, but avoiding construing it with *γ* in the Smoluchowski equation). Thus, we take the needs landscape as the potential, with *U*^‡^ and *U*^A^ becoming functions of thirst and hunger evaluated at the time of the previous reward collection $t={t}_{0}:{U}^{\ddagger }(T,H|g,s)$ and ${U}^{{\rm{A}}}(T,H|g,s)$, where the landscape *U*(*x*) specified in equation (1) depends internally on *s* and is scaled by multiplication with *g*. Following the harmonic approximation in (19) we approximate the well centres of our landscape as the log of the probability density function of the Gaussian at its mean (*μ*_w_ or *μ*_f_) and take the second derivative to get the frequencies *ν*:

20 $${\nu }_{{\rm{A}}}\approx \frac{1}{{\sigma }^{2}}$$

and

21 $${\nu }_{\ddagger }\approx \frac{2}{{\sigma }^{2}}$$

(note that we approximate the frequency of the transition state as double that of the source state well, a value approximately consistent with numerical evaluations).

Thus, we get the following expressions for the transition rates from food and from water, as a function of thirst and hunger:

22 $${\omega }_{{\rm{w}}{\rm{f}}}(T,H)\approx \left(\frac{\sqrt{\frac{1}{{\sigma }^{2}}\times \frac{2}{{\sigma }^{2}}}}{2\times \pi }\right)\times {{\rm{e}}}^{-\frac{{U}^{\ddagger }(T,H)-{U}^{w}(T,H)}{{n}^{2}}},$$

and

23 $${\omega }_{{\rm{f}}{\rm{w}}}(T,H)\approx \left(\frac{\sqrt{\frac{1}{{\sigma }^{2}}\times \frac{2}{{\sigma }^{2}}}}{2\times \pi }\right)\times {{\rm{e}}}^{-\frac{{U}^{\ddagger }(T,H)-{U}^{f}(T,H)}{{n}^{2}}}.$$

Plugging these equations into (18) and its corresponding expressions yields an equation for the transition probability of each behavioural trial pair, as a function of time, thirst magnitude, hunger magnitude, and the model parameters utilized in our equation of motion. This equation was used to evaluate theoretical predictions for Fig. 3h and Extended Data Fig. 7f. We note that this equation is only approximate and requires (1) that the potential well of the source state is deep relative to the transition state and (2) that the system follows a Boltzmann distribution at equilibrium^34^. Following requirement (2), we constrain the optimization by jointly optimizing both the transition probability of each reward pair observed in the experimental behavioural dataset, as well as the probability of all individual reward-choice trials derived from the Boltzmann distribution:

24 $$P\left({\rm{w}}| T,H\right)=\frac{\mathop{\int }\limits_{{x}_{{\rm{w}}}}\,{{\rm{e}}}^{-\frac{U(x,T,H)}{{n}^{2}}}}{\mathop{\int }\limits_{{x}_{{\rm{w}}}}\,{{\rm{e}}}^{-\frac{U(x,T,H)}{{n}^{2}}}+\mathop{\int }\limits_{{x}_{{\rm{f}}}}\,{{\rm{e}}}^{-\frac{U(x,T,H)}{{n}^{2}}}},$$

and

25 $$P\left({\rm{f}}| T,H\right)=\frac{\mathop{\int }\limits_{{x}_{{\rm{f}}}}\,{{\rm{e}}}^{-\frac{U\left(x,T,H\right)}{{n}^{2}}}}{\mathop{\int }\limits_{{x}_{{\rm{w}}}}\,{{\rm{e}}}^{-\frac{U\left(x,T,H\right)}{{n}^{2}}}+\mathop{\int }\limits_{{x}_{{\rm{f}}}}\,{{\rm{e}}}^{-\frac{U\left(x,T,H\right)}{{n}^{2}}}},$$

where *x*_w_ and *x*_f_ are the water and food zones of the landscape, respectively. These equations were used to evaluate theoretical predictions for Extended Data Fig. 7b,d. Finally, to additionally constrain the foraging weight parameter *s*, which scales the thirst and hunger magnitudes relative to ‘other needs’, we add to the joint optimization a Boltzmann-derived equation specifically for trials surrounding satiation, including misses (equations (24) and (25) do not consider misses and are evaluated on reward trials without flanking misses):

26 $$\begin{array}{l}P({\rm{o}}| T,H)=\frac{{\int }_{{x}_{{\rm{o}}}}{{\rm{e}}}^{-\frac{U(x,T,H)}{{n}^{2}}}}{{\int }_{x}{{\rm{e}}}^{-\frac{U(x,T,H)}{{n}^{2}}}};\\ P({\rm{w}}| T,H)=\frac{{\int }_{{x}_{{\rm{w}}}}{{\rm{e}}}^{-\frac{U(x,T,H)}{{n}^{2}}}}{{\int }_{x}{{\rm{e}}}^{-\frac{U(x,T,H)}{{n}^{2}}}};\\ P({\rm{f}}| T,H)=\frac{{\int }_{{x}_{{\rm{f}}}}{{\rm{e}}}^{-\frac{U(x,T,H)}{{n}^{2}}}}{{\int }_{x}{{\rm{e}}}^{-\frac{U(x,T,H)}{{n}^{2}}}},\end{array}$$

where the denominator is an integration over the entire landscape space and *x*_o_ is the ‘other needs’ zone of the landscape.

#### Loss function and model fitting computation

The above derivations yielded three sets of expressions for the probability of the experimental data as a function of the model and its parameters: (i) an expression for the transition probability between two sequential rewarded trials, dependent on time, thirst, hunger, and previous choice identity; (ii) an expression for the probability of a given reward, independent of time or previous choice but dependent on thirst and hunger; and (iii) an expression as in (i) but for trials near or during satiation, including misses. We then minimized the negative log-likelihood of the data across all experimental sessions under these theoretical model-derived equations by fitting the scale parameters *n*, *g* and *s* while leaving as fixed parameters the well centres and shapes for simplicity of interpretation (well centres are placed on an equilateral triangle and the standard deviation of each Gaussian well is the same). The data were partitioned into three sets, corresponding to (i), (ii) and (iii): sequential reward trial pairs as described above; individual rewarded trial outcomes and the thirst and hunger magnitudes at the time of each trial, excluding trials adjacent to misses; and individual Go-trial outcomes including misses near satiation (thirst and hunger <0.5). We separated the evaluation of non-sated (i, ii) and sated (iii) trials to avoid overfitting to early misses, which may also be due to errors in the behavioural task, as opposed to the mouse’s needs or goal. The joint loss function used was the average negative log-likelihood per trial in each of the three sets, added together. Because computing the Boltzmann-derived expressions used in (ii) and (iii) involved more computationally expensive numerical integrations, the trial data for (ii) and (iii) were batched to 1/50th the size of the entire dataset (~8,000 and ~11,000 trials, respectively). All equations were expressed using custom Python code utilizing the Jax^60^ library, enabling auto-differentiation, just-in-time compilation, auto-vectorization, and use of a corresponding optimization library Optax^69^ and the AdaBelief optimizer^70^ (with learning rate 10^−1^), which consumed the gradient of the joint loss function calculated with respect to *n*, *g* and *s*. Minimization of the loss function was performed until convergence.

For Fig. 3 and Extended Data Fig. 7, parameters for all analyses were fit using the procedure above on the entire behavioural dataset that was analysed in Fig. 1. For the optogenetic simulation experiment, an additional scale factor of 3.3 (obtained by grid search) was added to the gradient term *g* for best overlap with the experimental optogenetic stimulation results. We note that, since this experiment utilized a small subset of mice, the additional tuning of the gradient scale may account for animal-to-animal differences in these parameters not accounted for in the average values. For Fig. 4 and Extended Data Figs. 9 and 10, parameters were fit using the procedure above on the subset of mice used for Neuropixels recordings, and these same fit parameters were then used for all analyses, excluding the optogenetic analyses, in which the additional scaling factor of 3.3 obtained above was used.

### Simulations

Simulations of the mathematical model described above were implemented in custom Python code using the Jax library^60^ for just-in-time compilation (expediting simulations) and automatic differentiation (for landscape gradient calculations). The following fixed parameter set was used for all simulations: $${\mu }_{{\rm{o}}}=\left[\begin{array}{c}-8\\ 0\end{array}\right]$$, $${\mu }_{{\rm{w}}}=\left[\begin{array}{c}5\\ 7.5\end{array}\right]$$, $${\mu }_{{\rm{f}}}=\left[\begin{array}{c}5\\ -7.5\end{array}\right]$$, ${\sigma }^{2}\,=$ 20, *l* = 180, initial position $${x}_{0}=\left[\begin{array}{c}5\\ 0\end{array}\right]$$ (a saddle point between thirst and hunger wells), d*t* = 0.01, *r*_w_ = 0.006, and *r*_f_ = 0.004. For the analyses of Fig. 3c–h and Extended Data Fig. 7a–i, the *g*, *n* and *s* parameters were fit as described above to trial data from all behavioural sessions analysed in Fig. 1 and this single set of parameter values (*g* = 2.4383774, *n* = 2.74393, and *s* = 6.4874935) was used. For the behavioural optogenetic simulation analyses (Fig. 3j,k and Extended Data Fig. 7j–l), the same fit parameter values as above were used, except *g* was multiplied by a factor of 3.3 as described above. For the analyses of Fig. 4d–j and Extended Data Fig. 9e,h, parameters *g* and *n* were fit to trial data from the set of behavioural sessions of the Neuropixels recordings, as described above, and the single resulting set of parameters (*g* = 2.5563507, *n* = 2.807799, and *s* = 6.4874935) was used. For the optogenetic simulation analyses comparing simulated trajectories and neural data (Fig. 4m,o and Extended Data Fig. 10f), we used the same set of parameters as those used for Fig. 4d–j and Extended Data Fig. 9e,h, with *g* multiplied by a factor of 3.3 as described above. We note that in both sets of optogenetic analyses (behavioural and neural), qualitatively similar phenomena were observed with the base set of parameters (data not shown), but a closer quantitative match was observed with the additional scaling factor.

Each simulated run of an experiment with behavioural trials used a distinct randomly generated series of Go and No-Go trial times whose distribution in time matched the trial time distribution used in the actual behavioural assay. Simulations of Buridan’s assay (Fig. 3 and Extended Data Fig. 7) were run for 2 h of simulated time (720,000 steps with d*t* = 0.01 s). For visualization, simulations were run until a threshold of consecutive misses were observed, at which point the simulation was terminated; this threshold was set variably between 30–50 misses for visualization purposes. Simulations were initialized with ‘high thirst’ *T*_0_ values and ‘high hunger’ *H*_0_ values matching the initial values of experiment. Simulated sessions were run autonomously according to the above dynamics equations, parameters, initial conditions (*x*_0_, *T*_0_, *H*_0_) and trial times. A simulated dataset was composed of a set of simulated behavioural sessions matching the size of the corresponding experimental dataset. For analyses comparing the distribution of summary statistics in simulation to the summary statistics of experiment (Fig. 3), each dataset simulation was repeated 128 times with different random number generator keys, such that all simulated sessions contained a unique set of trial times and a unique session trajectory. In the case of model predictions of neural data, simulations of sessions were run for 1.5 h of simulated time (Fig. 4 and Extended Data Fig. 8) or 2 h or simulated time (Extended Data Fig. 9). For these analyses of model predictions, the number of simulated sessions was matched to the number of experimental sessions.

#### Simulations of behaviour with unbalanced noise to gradient scaling ratios

To illustrate the how the scaling terms on the gradient and the noise ($g$ and $n$) alter behavioural stickiness (Extended Data Fig. 7m,n), we ran model simulations with either a ‘too decoherent’ set of parameters (*g* = 2.0, *n* = 8.0 and *s* = 6.4874935) or a ‘too persistent’ set of parameters (*g* = 8.0, *n* = 0.5 and *s* = 6.4874935).

#### Simulations of optogenetic perturbation of behaviour

Simulations of hungry-only behaviour with optogenetic thirst perturbations were performed as described above, with the exception that the initial value of hunger *H*_0_ was set to 0.5 and the initial value of thirst *T*_0_ was set to 0.05 (Fig. 3i–k and Extended Data Fig. 7j–l). We note that the non-zero value of thirst tends to stochastically drive persistent water choices long after thirst perturbation as the system is more likely to remain in the water zone. Optogenetic thirst perturbations were modelled as a transient square wave, increasing the current value of ${\rm{T}}({\rm{t}})$ for the duration of the perturbation time (10 s, or 1,000 simulation steps) by a fixed ‘stimulation’ factor of 18 (modelling the detected thirst magnitude from optogenetic stimulation of osmotic thirst neurons as 4× that of a daily water restriction schedule; note however that hunger, thirst, and optogenetic thirst input are logarithmically related to the resulting landscape gradient magnitude). The effect of the simulated thirst perturbation on the energy landscape along the line between the centre of the food and water energy wells is shown in Fig. 3i. Optogenetic thirst stimulation in the context of hungry mice was simulated for 25 stimulation epochs across a 1-h-long simulated session with reward feedback set to 0 for simplicity of analysis. Simulated sessions were repeated to match the number of behavioural sessions in the corresponding experimental dataset.

#### Simulations of optogenetic perturbation prior to behaviour

We simulated optogenetic thirst perturbation experiments in hungry and thirsty mice in the absence of behaviour (Fig. 4k,m,o and Extended Data Fig. 10f). These simulations were performed as described above, but with initial values of hunger *H*_0_ set to 2.25 and thirst *T*_0_ set to 0.8 with additional optogenetic thirst input at 18 with duration 10 s. These simulated optogenetic perturbations were repeated 25 times with the same timing as actual experiments (optogenetic pulse onset every 1 min). The parameters were simulated for 3 simulation runs yielding 75 simulated thirst stimulation epochs.

#### Simulations of the forced-transition model

To assess an alternative model of choice dynamics (Extended Data Fig. 9) in which switches are driven by an external forcing function (as opposed to autonomously via the balance between noise and gradient), we preserved the energy landscape structure but modified the noise scale such that no switches occurred spontaneously over the duration of the simulation. This change has the effect of reducing our model to a multi-stable attractor system in which transitions wholly depend on external inputs to the system. We then incorporated a randomly occurring input force with magnitude sufficient to push the system from one landscape well to the other. We did not add noise to this force as it would reduce the number of successful transitions below that of the experimental data. In general, increasing the noise on the added force requires increasing the frequency of external-force events, such that this alternative model becomes less distinguishable from the diffusion landscape model we seek to compare it to.

#### Phase portraits

Phase portraits (Extended Data Fig. 9) for all simulated or experimental rewarded trials were generated using the current trial value of position along the goal dimension (simulated or measured in experimental data by projection of baseline activity onto a goal dimension fit by ridge regression, with regularization alpha parameter = 20) and the change in position along the goal dimension from trial to trial (which we define to be the velocity of activity along the goal dimension over a d*t* equal to the average time between trials). Trials were assigned to either stay or switch categories depending on whether the reward choice was the same as the previous reward (stay) or different (switch). Densities for stay and switch trials in the transition zone were quantified as described in the legend for Extended Data Fig. 9 and normalized to sum to 1. For model density quantifications, the simulated dataset trial data were resampled 1,000 times such that the number of stay and switch trials (across all simulated sessions) matched the fraction in the experimental dataset. This resampling controls for systematic bias in the quantified densities (Extended Data Fig. 9g,h) generated by stochastic differences in dataset switch rate.

### Statistics and reproducibility

Statistical parameters are described in legends. Box plots span lower and upper quartiles; lines indicate median values; whiskers, range of values within 1.5 times the interquartile range. Unless otherwise specified, confidence intervals were generated by bootstrapping.

### Reporting summary

Further information on research design is available in the Nature Portfolio Reporting Summary linked to this article.

## Online content

Any methods, additional references, Nature Portfolio reporting summaries, source data, extended data, supplementary information, acknowledgements, peer review information; details of author contributions and competing interests; and statements of data and code availability are available at 10.1038/s41586-023-06715-z.

### Supplementary information

Reporting Summary

Supplementary Video 1 Example model simulation of a session of Buridan’s assay. A forward simulation of a session of Buridan’s assay, generated by the neural-landscape diffusion model and corresponding to the simulated session visualized in Fig. 3c. Top, the video shows the current position in the needs subspace (white dot), the recent trajectory (squiggly white line), and the landscape depth (blue–green contour plot), all of which change across time according to the model dynamics (Extended Data Fig. 6). Middle, as Go-odour cues occur, food (orange bars), water (blue bars), or miss (no bar) choices are made according to which zone of the landscape the white dot is in. Bottom, the magnitude of thirst and hunger decrease after a short delay following water and food choices, respectively.

## Figures and Tables

**Extended Data Table 1:**
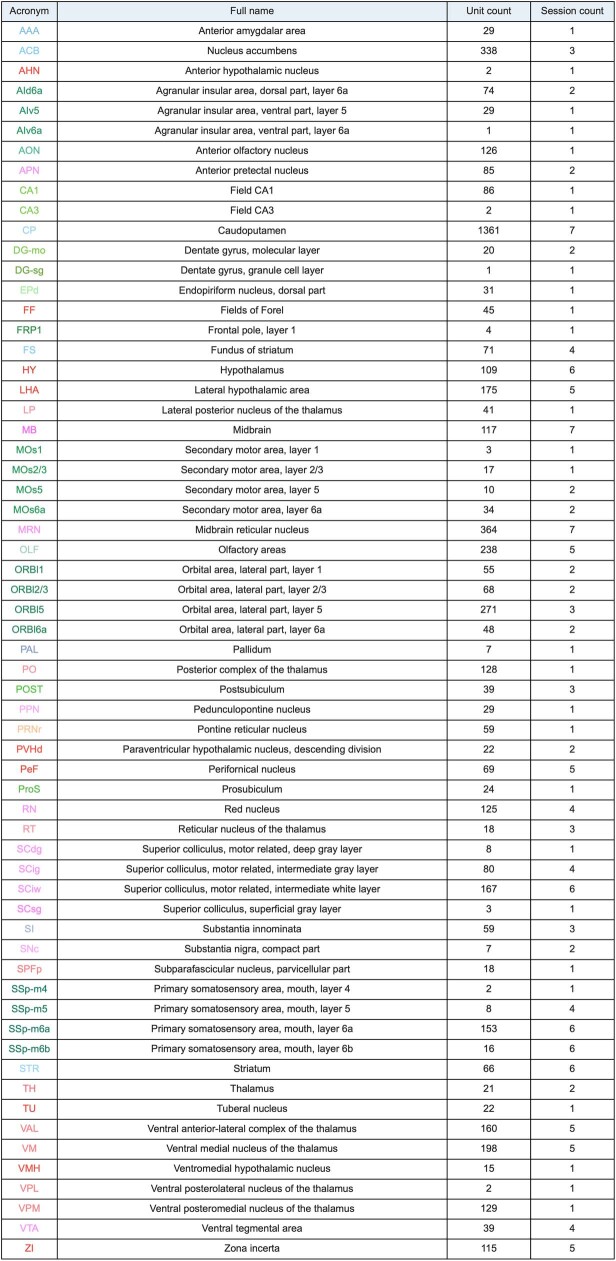
Allen Brain Atlas colors/abbreviations of brain regions and cell count for Neuropixels recordings

## Data Availability

The data from this study are available at 10.6084/m9.figshare.24153348.

## Code availability

Our Python code for working with datasets of multiple Neuropixels probes, pre-processed as described in the Methods, is available at https://github.com/erichamc/brainwide-npix and can be used to load the deposited data. Code for the neural landscape diffusion model and simulation is available at https://github.com/erichamc/neural-landscape-diffusion.
