# Supplementary material for: Neural landscape diffusion resolves conflicts between needs across time
Source: Nature. 2023 Nov 8;623(7987):571–9. doi: 10.1038/s41586-023-06715-z (PMC10651489; doi:10.1038/s41586-023-06715-z)
Supplement: Supplementary file 1 — Reporting Summary [file 41586_2023_6715_MOESM1_ESM.pdf]

nature portfolio

Corresponding author(s): Karl Deisseroth, Liqun Luo

Last updated by author(s): Sep 16, 2023

Reporting Summary

Nature Portfolio wishes to improve the reproducibility of the work that we publish. This form provides structure for consistency and transparency in reporting. For further information on Nature Portfolio policies, see our Editorial Policies and the Editorial Policy Checklist.

Statistics

For all statistical analyses, confirm that the following items are present in the figure legend, table legend, main text, or Methods section.

n/a	Confirmed	
☐	☒	The exact sample size (*n*) for each experimental group/condition, given as a discrete number and unit of measurement
☐	☒	A statement on whether measurements were taken from distinct samples or whether the same sample was measured repeatedly
☐	☒	The statistical test(s) used AND whether they are one- or two-sided<br>*Only common tests should be described solely by name; describe more complex techniques in the Methods section.*
☐	☒	A description of all covariates tested
☐	☒	A description of any assumptions or corrections, such as tests of normality and adjustment for multiple comparisons
☐	☒	A full description of the statistical parameters including central tendency (e.g. means) or other basic estimates (e.g. regression coefficient) AND variation (e.g. standard deviation) or associated estimates of uncertainty (e.g. confidence intervals)
☐	☒	For null hypothesis testing, the test statistic (e.g. *F*, *t*, *r*) with confidence intervals, effect sizes, degrees of freedom and *P* value noted<br>*Give P values as exact values whenever suitable.*
☐	☒	For Bayesian analysis, information on the choice of priors and Markov chain Monte Carlo settings
☒	☐	For hierarchical and complex designs, identification of the appropriate level for tests and full reporting of outcomes
☒	☐	Estimates of effect sizes (e.g. Cohen's *d*, Pearson's *r*), indicating how they were calculated

Our web collection on statistics for biologists contains articles on many of the points above.

Software and code

Policy information about availability of computer code

Data collection	Matlab 2019, Bpod Gen_2, custom Matlab code, Spike GLX versions 2 and 3
Data analysis	Data preprocessing: CatGT 3.3, TPrime 1.7, C_Waves 2.0, Kilosort 3.0, ecephys_spike_sorting 0.2, Phy2. Data analysis: Python code including the following open source libraries: Jupyter (1.0.0), IPython (8.9.0), Numpy (1.23.5), Scipy (1.10.0), Pandas (1.5.3), Scikit-learn (1.2.1), Jax (0.4.6), Matplotlib (3.4.2), Seaborn (0.12.2), Statsmodels (0.13.0), Scanpy (1.9.3), AllenSDK (2.15.1). Custom Python code related to loading, preprocessing, and plotting spike-sorted Neuropixels, anatomical, and event data is available at https://github.com/erichamc/brainwide-npix. Custom Python code related to the neural landscape diffusion model and simulation is available at https://github.com/erichamc/neural-landscape-diffusion.

For manuscripts utilizing custom algorithms or software that are central to the research but not yet described in published literature, software must be made available to editors and reviewers. We strongly encourage code deposition in a community repository (e.g. GitHub). See the Nature Portfolio guidelines for submitting code & software for further information.

Data

Policy information about availability of data

All manuscripts must include a data availability statement. This statement should provide the following information, where applicable:

- Accession codes, unique identifiers, or web links for publicly available datasets
- A description of any restrictions on data availability
- For clinical datasets or third party data, please ensure that the statement adheres to our policy

The data from this study are available at https://doi.org/10.6084/m9.figshare.24153348.

Human research participants

Policy information about studies involving human research participants and Sex and Gender in Research.

Reporting on sex and gender	N/A
Population characteristics	N/A
Recruitment	N/A
Ethics oversight	N/A

Note that full information on the approval of the study protocol must also be provided in the manuscript.

Field-specific reporting

Please select the one below that is the best fit for your research. If you are not sure, read the appropriate sections before making your selection.

☒ Life sciences ☐ Behavioural & social sciences ☐ Ecological, evolutionary & environmental sciences

For a reference copy of the document with all sections, see nature.com/documents/nr-reporting-summary-flat.pdf

Life sciences study design

All studies must disclose on these points even when the disclosure is negative.

Sample size	No sample-size calculation was used to pre-determine sample sizes. Sample sizes were chosen based on prior experience in our labs.
Data exclusions	The following criteria were used to determine whether an animal was included in the analysis: (1) The behavioral task was learned adequately, with >90% responses withheld during No-Go odors; >90% responses withheld during the 1s before odor onset; and at least 30 rewards collected for each of food and water when the behavioral assay was conducted following food and water restriction; (2) Electrophysiological recordings had minimal electrical artifacts or other sources of noise; (3) In the case of optogenetic manipulations, animals were pre-screened for responsiveness to thirst stimulation when sated in their homecage (in some animals, inadequate viral expression in the target region resulted in non-responsiveness to thirst stimulation in homecage and thus led to subject exclusion).
Replication	Each animal in the study is an independent experimental replicate performed separately. Results were consistent across animals. Efforts to replicate the main phenomena described in this work using a freely moving version of the behavioral assay also succeeded (see Extended Data Figure 1).
Randomization	Subject randomization by genotype was not applicable, as there was only a single experimental genotype group for each experiment.
Blinding	Experimenters were not blinded to experimental conditions during data collection, though data collection was performed automatically. Experimenters were blinded to experimental conditions during data analysis.

Reporting for specific materials, systems and methods

We require information from authors about some types of materials, experimental systems and methods used in many studies. Here, indicate whether each material, system or method listed is relevant to your study. If you are not sure if a list item applies to your research, read the appropriate section before selecting a response.

Materials & experimental systems

n/a	Involved in the study
☒	☐ Antibodies
☒	☐ Eukaryotic cell lines
☒	☐ Palaeontology and archaeology
☐	☒ Animals and other organisms
☒	☐ Clinical data
☒	☐ Dual use research of concern

Methods

n/a	Involved in the study
☒	☐ ChIP-seq
☒	☐ Flow cytometry
☒	☐ MRI-based neuroimaging

Animals and other research organisms

Policy information about studies involving animals; ARRIVE guidelines recommended for reporting animal research, and Sex and Gender in Research

Laboratory animals	C57BL/6J mice aged 6-24 weeks; C57BL/6J mice aged 6-24 weeks; Rxfp1 em1(cre)Ngai (Rxfp1-2A-Cre) mice aged 6-24 weeks. Animals were maintained on a reverse light-dark cycle and standard housing conditions (18-23ºC, 40-60% humidity).
Wild animals	The study did not involve wild animals.
Reporting on sex	Only female mice were used in this study.
Field-collected samples	The study did not involve samples collected in the field.
Ethics oversight	All procedures involving mice followed the animal care guidelines approved by Stanford University's Administrative Panel on Laboratory Animal Care.

Note that full information on the approval of the study protocol must also be provided in the manuscript.
